# Heterogeneity in the Effectiveness of Non-pharmaceutical Interventions During the First SARS-CoV2 Wave in the United States

**DOI:** 10.3389/fpubh.2021.754696

**Published:** 2021-11-29

**Authors:** William K. Pan, Daniel Fernández, Stefanos Tyrovolas, Giné-Vázquez Iago, Rishav Raj Dasgupta, Benjamin F. Zaitchik, Paul M. Lantos, Christopher W. Woods

**Affiliations:** ^1^Nicholas School of Environment, Duke University, Durham, NC, United States; ^2^Duke Global Health Institute, Duke University, Durham, NC, United States; ^3^Serra Húnter Fellow, Department of Statistics and Operations Research, Polytechnic Universitat Politècnica de Catalunya BarcelonaTech (UPC), Barcelona, Spain; ^4^Institute of Mathematics of UPC - BarcelonaTech (IMTech), Barcelona, Spain; ^5^WHO Collaborating Centre for Community Health Services (WHOCC), School of Nursing, The Hong Kong Polytechnic University, Hong Kong, Hong Kong SAR, China; ^6^Parc Sanitari Sant Joan de Déu, Universitat de Barcelona, Fundació Sant Joan de Déu, Barcelona, Spain; ^7^Instituto de Salud Carlos III, Centro de Investigación Biomédica en Red de Salud Mental (CIBERSAM), Madrid, Spain; ^8^Department of Earth and Planetary Sciences, Johns Hopkins University, Baltimore, MD, United States; ^9^Duke School of Medicine, Duke University, Durham, NC, United States

**Keywords:** SARS-CoV-2, non-pharmaceutical intervention (NPI), doubling time, mortality rate, United States

## Abstract

**Background:** Attempts to quantify effect sizes of non-pharmaceutical interventions (NPI) to control COVID-19 in the US have not accounted for heterogeneity in social or environmental factors that may influence NPI effectiveness. This study quantifies national and sub-national effect sizes of NPIs during the early months of the pandemic in the US.

**Methods:** Daily county-level COVID-19 cases and deaths during the first wave (January 2020 through phased removal of interventions) were obtained. County-level cases, doubling times, and death rates were compared to four increasingly restrictive NPI levels. Socio-demographic, climate and mobility factors were analyzed to explain and evaluate NPI heterogeneity, with mobility used to approximate NPI compliance. Analyses were conducted separately for the US and for each Census regions (Pacific, Mountain, east/West North Central, East/West South Central, South Atlantic, Middle Atlantic and New England). A stepped-wedge cluster-randomized trial analysis was used, leveraging the phased implementation of policies.

**Results:** Aggressive (level 4) NPIs were associated with slower COVID-19 propagation, particularly in high compliance counties. Longer duration of level 4 NPIs was associated with lower case rates (log beta −0.028, 95% CI −0.04 to −0.02) and longer doubling times (log beta 0.02, 95% CI 0.01–0.03). Effects varied by Census region, for example, level 4 effects on doubling time in Pacific states were opposite to those in Middle Atlantic and New England states. NPI heterogeneity can be explained by differential timing of policy initiation and by variable socio-demographic county characteristics that predict compliance, particularly poverty and racial/ethnic population. Climate exhibits relatively consistent relationships across Census regions, for example, higher minimum temperature and specific humidity were associated with lower doubling times and higher death rates for this period of analysis in South Central, South Atlantic, Middle Atlantic, and New England states.

**Conclusion and Relevance:** Heterogeneity exists in both the effectiveness of NPIs across US Census regions and policy compliance. This county-level variability indicates that control strategies are best designed at community-levels where policies can be tuned based on knowledge of local disparities and compliance with public health ordinances.

## Introduction

During the first COVID-19 wave (15th January to 31th May 2020), the disease spread rapidly across the globe, infecting over 3 million people with the SARS-CoV-2 virus. One-third of all reported cases and one-quarter of all deaths were in the United States. Public health interventions to reduce the spread of COVID-19 in the US varied in timing and level of aggressive measures undertaken. No official US policy existed to prevent COVID-19 transmission until January 31, 2020, when a presidential order blocked the entry of non-US citizens into the US traveling from China. The state of Washington declared the first State of Emergency (February 29), followed by California (March 4) and Maryland (March 5); however, many counties implemented restrictive policies prior to state action. For example, three counties in Washington (King, Pierce, Snohomish) and four in Arkansas (Grant, Jefferson, Pulaski, Saline) ordered school closings on March 12 vs. statewide closures on March 17; counties in Pennsylvania, California, and New Jersey closed non-essential businesses prior to the state; and counties in California and Idaho issued restrictions on mass gatherings before state policies.

Early action by counties was motivated by both the ability to legislate response and the multifaceted issues that impact disease risk at local levels. Such issues include racial disparities, with a disproportionate number of Black and Hispanic Americans reported to be infected or dying (1–3), urban vs. rural characteristics that influence transmission and policy implementation (4), and disease spillover from neighboring counties, which can be exacerbated by economic disparities and shared environmental risks (5). There is growing consensus that SARS-CoV-2 is an airborne pathogen, spread primarily through respiratory aerosols (6–8) that can be influenced by both micro- and macro-environments. Much work has focused on indoor micro-environments to help understand superspreading events, such as in restaurants, call centers, and large social gatherings (9–11). The relationship between COVID-19 transmission and macro-environmental factors is more difficult, with early work evaluating relative humidity (RH), temperature, and UV exposure; however, findings have varied with some studies finding no effect (12), inverse relationships (13), or mixed effects (14, 15). Elevated humidity has been associated with an increase in organic aerosols and higher levels of small particulate air pollutants (PM2.5, PM10, and O_3_) (16, 17), which have been correlated with transmission (18, 19); however, the simultaneous use of RH and temperature in models of viral respiratory illness is not recommended due to their direct functional relationship (20).

Among other factors, the absence of vaccines during the COVID-19's first wave resulted in the application of non-pharmaceutical interventions (NPI, also known as community mitigation strategies) to control COVID-19. Unfortunately, without a randomized trial to quantify the effect size of NPIs or the potential causes of heterogeneity across different regions of the US, policymakers relied on modeling studies and early evidence from Asia to guide decisions. These included susceptible-exposed-infectious-recovered (SEIR) models attempting to quantify the effects of isolation and contact tracing (21) and how varying NPI effectiveness may influence demands on critical care resources (22); and studies in Wuhan and Hong Kong reporting how strict interventions (i.e., quarantine, social distancing, shelter in place and active case detection) reduced the COVID-19 reproductive number (R_0_) (23, 24). Over the past year, several studies have attempted to quantify NPI effects, with a recent meta-analysis summarizing studies from around the world that quantified how various strategies helped contain COVID incidence (25). In the US, the majority of studies trying to quantify NPI effects have focused on the state-level (i.e., state-level policies impacting state-level cases and deaths) (26–29). For example, White and Hebert-Dufresne (27) analyzed five state-level policy effects, finding that only restaurant restrictions significantly correlated with higher doubling times; Chernozhukov et al. (26) study four state policies using a structural equation modeling approach, showing important effects of masking, business closures, and stay-at-home orders on both cases and deaths; and Auger (28) studied the effect of statewide school closures on COVID-19 cases and deaths using a negative binomial interrupted time series analysis, finding a 62% decline in cases and 58% decline in mortality following school closure. However, these studies ignore county-level heterogeneity and restrict their analyses to a specific set of policies without considering the joint effect of two policies implemented simultaneously. Aggregating county-level data ignores important social determinants as well as the reliance on county governments by states to make essential decisions regarding policy implementation and enforcement that explain variations in NPI effectiveness (29, 30). The most comprehensive county-level analysis to date in the US was conducted by Ebrahim et al. (30), who found both widespread county-level variation in policies and identified business closures as having the most important effect on COVID-19 cases; however, this study was limited to just one-third of US counties and relied on the estimation of R0 rather than using reported cases.

The goal of this study is to evaluate national and sub-national effects of the four levels of NPIs using county-level data on policies, cases and socio-environmental factors during the first wave in the US until implementation to Phase 1 reopening (lifting of policies), if specified, otherwise to May 29. We leverage the phased implementation of policies at the county level, using a stepped-wedge cluster-randomized trial (SW-CRT) framework (31). We quantify NPI effects on COVID-19 daily case incidence, doubling time, and reported deaths across nine US Census regions.

## Methods

### Outcome Data

Multiple data sources were used to confirm county-level daily cases and deaths from SARS-CoV2 infection. Data from the Johns Hopkins University Center for System Science and Engineering Coronavirus Resource Center (JHU-CSSE, https://coronavirus.jhu.edu/) were compared to county data reported on state health department's websites, using the state data when discrepancies were noted (i.e., counties from 34 states using the JHU-CSSE data were discrepant with state-reported county data). In addition, any county whose cumulative cases or deaths declined over time was flagged and adjusted using state- or county data. The final data are counties from all 50 US states and the District of Columbia extending from January 22 through May 29, 2020. US territories (American Samoa, Guam, the Northern Mariana Islands, Puerto Rico, and the U.S. Virgin Islands) were excluded.

### Policy Data

The effective date of each public health intervention and the phased reopening at the state and county levels was initially extracted from online policy databases (32–36). For discrepancies or missing county policies, we obtained policy dates in two steps: (1) searching the county's state government website for reported county policies; and (2) if state websites did not report county policies or if the county reported at least one COVID-19 case prior to the issuance of any state order, we conducted a systematic search of gray literature for each county's policies, focusing on county websites (if existent) and local news websites. We categorized 12 policies into 4 levels of disease control following the New Zealand alert system and Oxford classification (37, 38): Level 1 (low)—governor declaration of a State of Emergency; Level 2 (moderate)—school closures, restricting access (visits) to nursing homes, or closing restaurants and bars; Level 3 (high) – non-essential business closures, suspending non-violent arrests, suspending elective medical procedures, suspending evictions, or restricting mass gatherings of at least 10 people; and Level 4 (aggressive)—sheltering in place / stay-at-home, public mask requirements, or travel restrictions. These levels are mostly cumulative, meaning counties tended to implement policies sequentially and jointly, for example, 67% of counties implemented almost all level 3 policies at the same time, while 23% implemented them within 7 days of their initial level 3 policy. Note that our initial analysis found no effect of the two federal policies blocking entry to the US for non-US citizens (i.e., from China issued January 31 and from Schengen European countries issued March 11) on COVID-19 morbidity or mortality propagation; thus, we classified them as the “non-intervention” period. Finally, NPI effects were measured up to 5 days after county reopening, defined as opening non-essential businesses (with capacity restrictions), allowing public gatherings of more than 10 people, opening public spaces, or easing shelter in place orders. All but 12 states entered some phase of reopening by May 29 and six states allowed counties to open at their discretion (CA, IA, MD, NE, OR, WY).

Policy compliance was measured by comparing the number of trips recorded in each county from 2020 to 2019 based on data from the Maryland Transportation Institute and Center for Advanced Transportation Technology (https://data.bts.gov/Research-and-Statistics/Trips-by-Distance/w96p-f2qv). Several studies have demonstrated the utility of mobility data as proxy measures of policy compliance (30, 39–41). Using results from Nouvellet et al. (42), we defined gradients of mobility decline associated with reductions in R0. A four-level variable for compliance was created, where non-compliance (level 0) was defined as a mobility difference from 2019 to 2020 of <15%, low compliance (level 1) as a decline in mobility of 15–30%, moderate compliance (level 2) as a decline of 30–50% and high compliance (level 3) as mobility declines >50%. We then summed the time-lagged 10-day compliance and divided by maximum compliance (30) to create a scaled measure, from 0 to 10, representing the average level of policy compliance over the past 10 days. A value closer to 10 indicates high compliance (low mobility compared to 2019), while values closer to zero indicate lower compliance (more mobility). We expect to see values close to zero prior to the initiation of polices and as policies were removed.

### Demographic and Environmental Data

Demographic data are from the US Census Bureau. This includes county-level age, sex and racial composition, migration, and educational data from the 2018 American Community Survey (43), land area to compute population density (1,000 people per square-km) (44), and poverty (45). Analyses were stratified by the nine US Census regions (Pacific, Mountain, West North Central, East North Central, West South Central, East South Central, South Atlantic, Middle Atlantic, New England) to evaluate differential policy effects.

We use the USDA Rural-Urban Continuum Code that categorizes counties into nine levels of rural-urban characteristics (46). Three levels indicate metropolitan areas of (1) 1 million or more people, (2) 250,000 to 1 million people, and (3) fewer than 250,000 people. Four urban levels classified by size and adjacency to a metropolitan area: (4) 20,000 or more people adjacent; (5) 20,000 or more, not adjacent; (6) 2,500 to 19,999, adjacent; and (7) 2,500 to 19,999, not adjacent. And two rural levels: (8) <2,500 population, adjacent to a metropolitan area; and (9) <2,500 population, not adjacent.

Environmental data are from the North American Land Data Assimilation System (NLDAS). The NLDAS provides several daily hydrometeorological measures, for which we define 10-day temporal lags of minimum air temperature (Celsius), specific humidity (g/kg) and bias-corrected shortwave radiation (W/m^2^).

### Statistical Methods

Outcomes were defined at the county level as the number of new daily cases, new deaths, and case doubling time. Doubling time is the number of days required to double the cumulative case count on a particular day. Policy levels were time-varying, coded as a 1 while a level was active and 0 otherwise, and as the number of days since an intervention level was initiated.

Descriptive differences in policy implementation were examined using Chi-square and *t*-tests. To evaluate intervention levels and socio-environmental factors, negative binomial mixed models were fit using an approach similar to a Hussey and Hughes SW-CRT analysis (47). The model is specified as follows:


$$\begin{array}{l} \ln\left(y_{it}\right)=\ln\left(N_{i}\right)+x_{0}\beta_{0}+x_{1}\beta_{1}+\ldots x_{p}\beta_{p}+u_{i} \end{array}$$

Where *y*_*it*_ is the outcome (cases, deaths, doubling time) for county *i* on study day *t*; ln(*N*_*i*_) is the offset where *N*_*i*_ is the population density for county *i*; parameters *x*_*p*_β_*p*_ represent the fixed predictors (*x*_*p*_) and their associated parameters; and *u*_*i*_ is the random county effect. For the new cases and death models, we use population density as the offest, but no offset is included for doubling time. In addition, for case and death models, period (study day) is included as a continuous variable while the doubling time model uses period as a categorical variable (similar to the SW-CRT modeling approach). When entered as a continuous variable, we evaluated inclusion of linear and quadratic period terms. Each outcome was fit for the country as a whole and separately for each US Census region, combining East and West North Central states, East and West South Central states, and Middle Atlantic and New England states. Finally, to test policy effects, we evaluate the time-varying policy variables, i.e., duration of intervention, which are entered into case and doubling time models simultaneously, but as individual time-varying policy effects in death rate models (i.e. comparing policy 2 vs. 1 or nothing, policy 3 vs. 0–2, etc.). Since there are four policies, we use the Holm-Bonferroni multiple comparisons correction to evaluate significance (48).

Case and doubling time models were identified using one randomly selected census region and then evaluating model fit using AIC (49). Variables considered include: rural-urban continuum code; minority (Black and Hispanic) and total population density; net county migration rate in 2018; percent of the 2018 county population Black (alone or mixed race), Hispanic (alone or mixed race), living in poverty, or with a college education or higher; and climate parameters (10-day lags for minimum temperature, specific humidity, and UV radiation). Once a final model was determined, it was used to fit to all regions combined (adding an indicator for census region) and for each individual census region. Final models are shown in the full regression tables (Supplementary Table B0 for the country as a whole; Supplementary Tables B1, C1, D1 for COVID-19 models of cases, doubling time, and deaths by Census region, respectively). Climate variables were evaluated only using doubling time and death outcomes. Final models were fit using SAS 9.4 with Gaussian adaptive quadrature.

Secondary to our evaluation of policy effects, we use the above models to describe social and environmental disparities observed across Census regions. In addition, we conduct an analysis on factors associated with policy compliance by comparing change in county compliance during the first 15 and 30 days of March to socioeconomic and environmental characteristics. Therefore, since we define compliance as a 10-day lagged sum of compliance, we fit two models to estimate factors associated with compliance change from March 1 to 15 (i.e., change compliance from April 21-March 1 to March 5–15) and change from March 1 to 30. The compliance model includes random county effects within states and estimates both standardized and non-standardized beta coefficients. Standardized Beta coefficients only standardize predictor variables, not the response; therefore, the interpretation of the standardized beta is a one standard deviation unit change in a covariate being associated with a change in compliance. We note that on March 1, 98.8% of counties did not have any COVID policies; by March 15, 50.9% had enacted at least policy level 2; and by March 30, 98.9% of counties established policy level 3 or 4.

## Results

We obtained complete data from 3,142 counties. 339 counties (10.8%) from 26 states created policies prior to their state government, the majority (211 or 62%) were located in Texas, Nebraska, Missouri, and Pennsylvania. Counties initiating policies prior to the state were more likely to be located in metropolitan areas (17.5% metro counties vs. 6.8% non-metro countries adopted early policies, *p* < 0.001), have populations with higher educational levels, higher percentages of Hispanic population, and fewer people living in poverty (respectively, counties with early policy adoption had 26.3, 15.9, and 13.8% of their populations with a college degree, Hispanic descent [mixed or alone], and living in poverty, vs. 21.0, 8.5, and 15.3% for countries that did not adopt early policies, *p* < 0.001).

**Figure 1:**
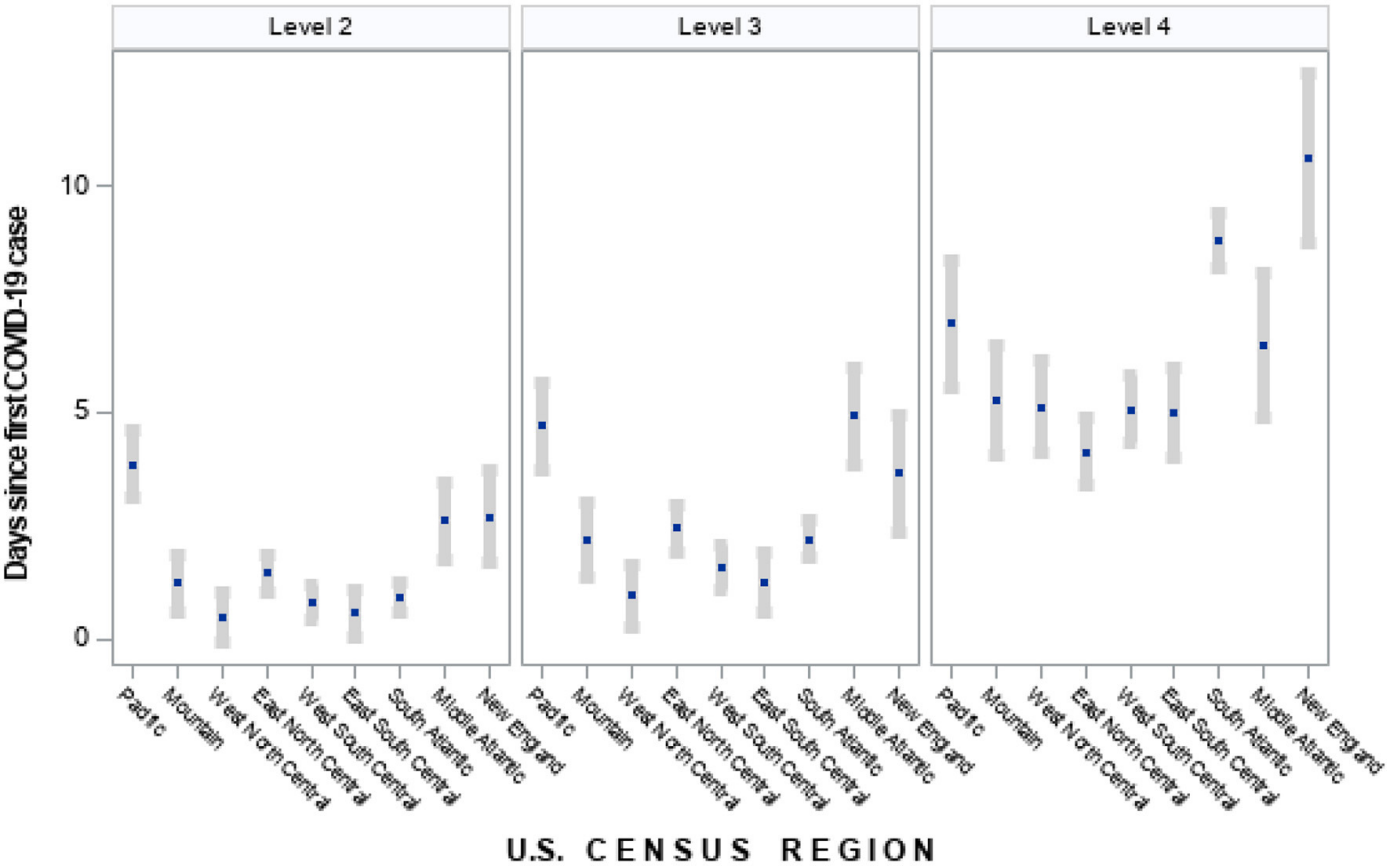
Time between first COVID-19 case to policy initiation by US census region (mean days and 95% confidence interval). Policies classified under each level of intervention: Level 2—school closures, restricting access to nursing homes, closing restaurants and bars; Level 3—non-essential business closures, suspending non-violent arrests, suspending elective medical procedures, suspending evictions, restricting mass gatherings of at least 10 people; Level 4—sheltering in place/stay-at-home, mask requirements in public, travel restrictions. States classified for each region are as follows: Pacific—CA, WA, OR, HI, AK; Mountain—MT, WY, ID, NV, UT, CO, AZ, NM; West North Central—ND, SD, NE, KS, MN, IA, MO; East North Central—WI, IL, MI, IN, OH; West South Central—TX, OK, AR, LA; East South Central—KY, TN, MS, AL; South Atlantic—FL, GA, SC, NC, VA, WV, DC, MD, DE; Middle Atlantic—NY, PA, NJ; New England—ME, CT, NH, MA, CT, RI.

Speed of policy adoption varied, with fewer days spanning initial case detection and initiation of NPIs in counties located in North and South Central states, while counties in New England, Middle Atlantic, and Pacific states had more days between initial case detection and policy initiation (Figure 1). However, in these later three regions, the first COVID-19 case was detected an average of 10 days prior to North and South Central states. Counties in these three regions (Pacific, Middle Atlantic and New England) had the longest duration of level 1 policies, but the shortest duration of time from initial case detection to the most strict (level 4) policies (Table 1). County and state governments in New England and Mountain states were more likely to initiate policies before the first reported COVID-19 death compared to other regions (data not shown). Once initiated, policy duration averaged 5.7, 3.6, 11.9, and 44.3 days for levels 1–4, respectively, with significant variation by state and some states having zero days for any particular level (Supplementary Table A1, Table 1).

**Table 1:** Median policy duration by US census region.

**Census region**	**Level 1**	**Level 2**	**Level 3**	**Level 4**
	**Median (IQR)**	**Median (IQR)**	**Median (IQR)**	**Median (IQR)**
Pacific	11 (8–13)	2 (1–3)	3 (0–6)	33 (14–52)
Mountain	4 (4–5)	4 (1–4)	6 (0–8)	30 (11–48)
North Central	6 (5–7)	4 (2–4)	8 (4–14)	23 (0–45)
South Central	6 (4–6)	2 (1–5)	9 (5–13)	31 (14–47)
South Atlantic	5 (4–6)	4 (1–8)	10 (6–15)	27 (10–44)
Middle Atlantic & New England	9 (5–10)	3 (1–4)	2 (0–5)	34 (15–52)

**Figure 2:**
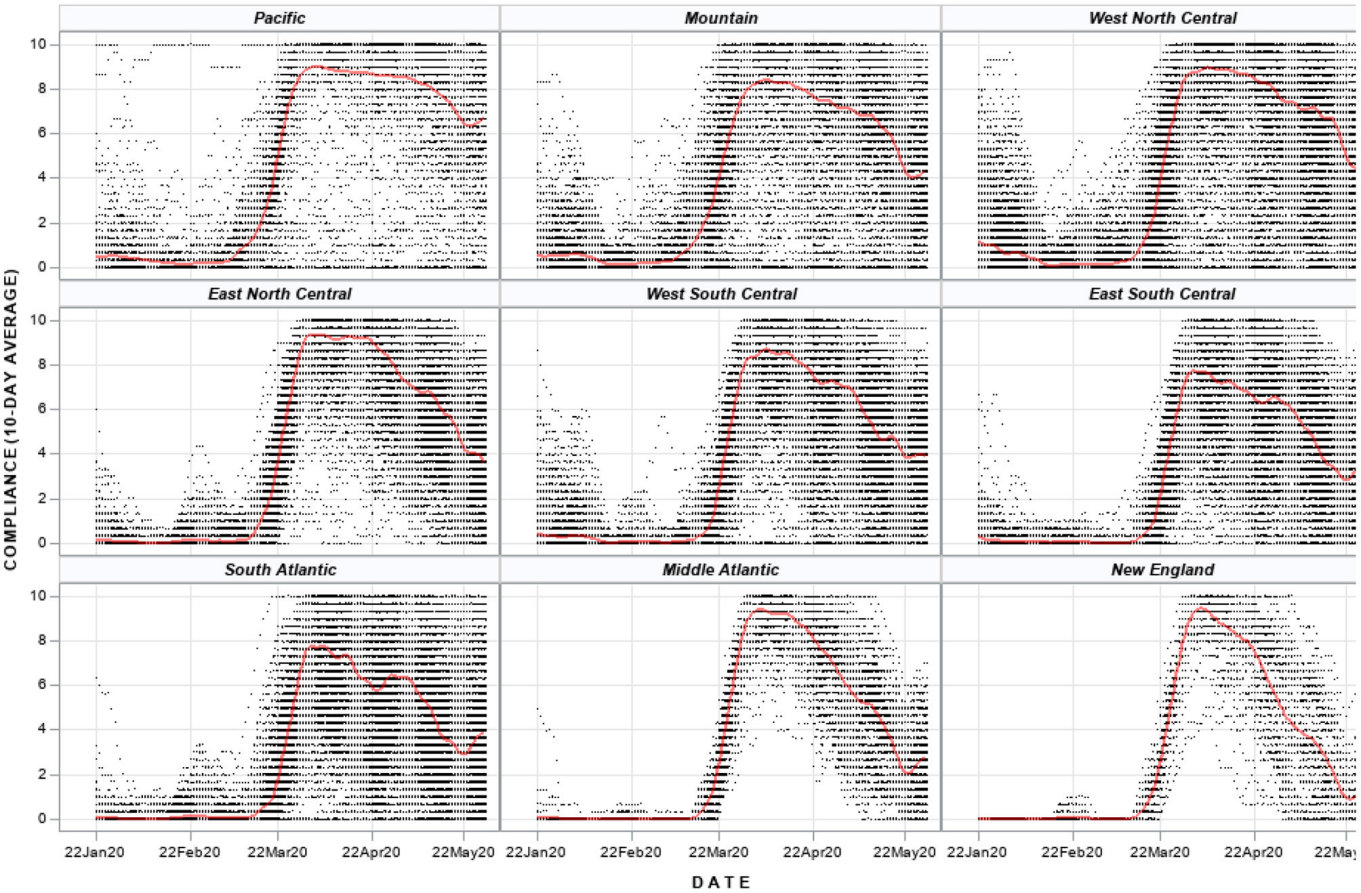
Compliance with policy interventions for US counties by census region. Each dot represents a county in the US and each county has a daily time series of compliance. The red line is a fitted penalized b-spline. US Census Regions: Pacific—CA, WA, OR, HI, AK; Mountain—MT, WY, ID, NV, UT, CO, AZ, NM; West North Central—ND, SD, NE, KS, MN, IA, MO; East North Central—WI, IL, MI, IN, OH; West South Central—TX, OK, AR, LA; East South Central—KY, TN, MS, AL; South Atlantic—FL, GA, SC, NC, VA, WV, DC, MD, DE; Middle Atlantic—NY, PA, NJ; New England—ME, CT, NH, MA, CT, RI.

Compliance with policies was highly variable. As expected, prior to the initiation of most policies (January and February 2020), compliance measures were low (i.e., no policies to follow). Most states and counties initiated strict policies in March 2020, which is reflected by the sharp increase in compliance in most regions during this month (Figure 2). In Middle Atlantic and New England States, there was high, homogenous compliance with few counties reporting moderate changes to mobility; however, counties in Mountain, West North Central, East South Central and South Atlantic exhibited high heterogeneity in compliance. Even within states, considerable heterogeneity was observed; for example, four states did not have any counties implement level 4 policies: Iowa, Nebraska, North Dakota and South Dakota. Among these four, policy compliance increased rapidly, but only counties in Iowa maintained high and relatively homogenous policy compliance across the state (Supplementary Table A1, Supplementary Figure A1). Similarly, four states had over 70 days with a level 4 policy (California, Hawaii, Illinois, New Jersey) with three experiencing high, homogenous compliance across the state and one (Illinois) having increasing heterogeneity in compliance over time (Supplementary Figure A1).

### Policy Effects on COVID-19 Propagation and Mortality

#### Case Rates

**Figure 3:**
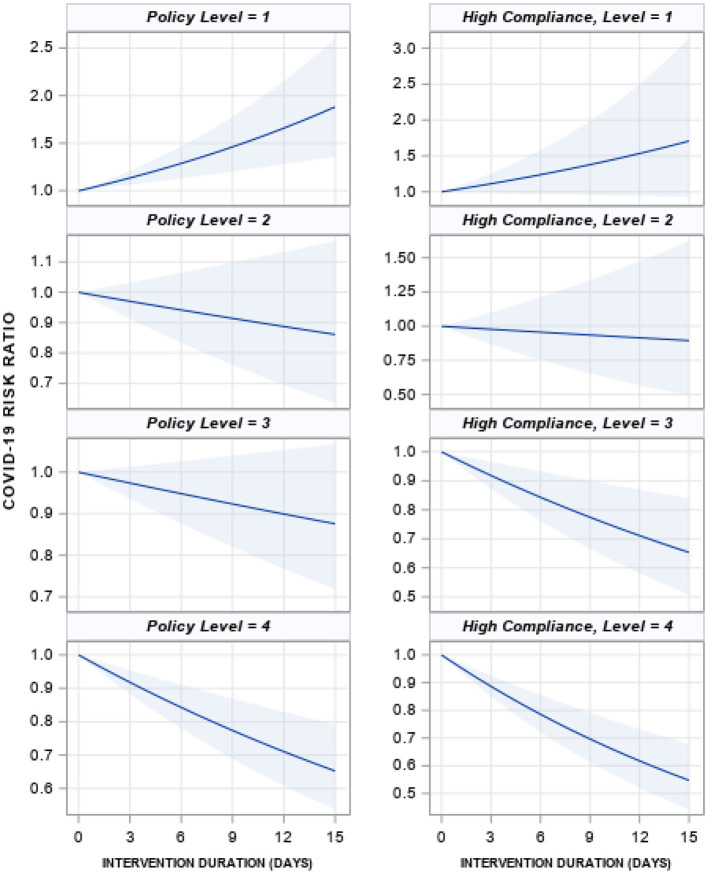
Mean effect of each policy level on the COVID-19 case rate if the policy was implemented for 15 days, low and high compliance. The solid blue line is the mean effect size for each intervention with 95% confidence intervals (shaded blue). A value of 1 represents no change, 1.5 is a 50% increase in cases and 0.5 is a 50% decrease. The left column is for low compliance (no change in behavior in 2020 compared to 2019, scaled value 0) and the right is for high compliance (scaled value 8). Effect sizes are from negative binomial models reported in Supplementary Table B0.

Model results for the country as a whole indicate that, under conditions of high policy compliance (scaled value of 8), intervention level 4 achieved a 50% reduction in COVID-19 case rates in 16 days (95% CI for 16-day case reduction: 40.2–61.8%), compared to 22 days for intervention level 3 (95% CI: 35.1–71.5%, Figure 3, Table 2). Intervention levels 1 and 2 never achieve significant reductions in case rates; in fact, longer duration of level 1 policies was positively associated with cases. Under conditions of low compliance (scaled value of zero), only duration of level 4 policies achieved a significant decline, while level 1 policies were inversely associated with COVID-19 cases. The time needed for level 4 policies to achieve a 50% decline increased to 20 days under these conditions.

**Table 2:** Model predicted policy effects on case rates, doubling time, and death rates for all US counties.

	**Case rates**		**Doubling time**		**Death rates**	
	**Log beta**	**(95% CI)**	**Log beta**	**(95% CI)**	**Log beta**	**(95% CI)**
**Fixed intervention effect**
Level 1	0.768	(0.56–0.97)^†††^	0.059	(−0.03–0.15)	2.371	(1.18–3.57)^††^
Level 2	1.373	(1.14–1.6)^†††^	0.082	(−0.03–0.19)	2.999	(1.8–4.2)^†††^
Level 3	1.516	(1.28–1.75)^†††^	0.072	(−0.04–0.19)	3.187	(2–4.38)^†††^
Level 4	1.707	(1.47–1.94)^†††^	0.143	(0.03–0.26)^†^	3.539	(2.35–4.72)^†††^
**Duration of intervention effect**
Level 1	0.03	(0.01–0.05)^††^	0.004	(−0.01–0.01)	−0.018	(−0.05–0.01)
Level 2	−0.018	(−0.04–0)	0.012	(0–0.02)^†^	0.028	(0–0.05)^†^
Level 3	−0.012	(−0.02–0)	0.011	(0–0.02)^†^	0.062	(0.04–0.08)^†††^
Level 4	−0.032	(−0.04–0.02)^†††^	0.020	(0.01–0.03)^†††^	0.056	(0.04–0.07)^†††^

††† *p < 0.0001*,

†† *p < 0.01*,

† *p < 0.05*.

*The log beta values represent the log change in the outcome when an intervention policy is in place or the change for the number of days the policy is in effect. Results are from three negative binomial models using all counties from the US (Supplementary Table B0). Case and Doubling Time Models included the same covariates, except for the addition of population density in the Doubling Time Model. The model for Death Rates was reduced to ease model fit*.

Policy effects were variable across US Census regions (Table 3). For an average level of policy compliance (scaled value of 6), intervention level 4 was significantly associated with declines in COVID-19 case rates in Pacific and Mountain regions, yet did not achieve significance in other regions (Table 3, Supplementary Figure B1, Supplementary Table B1). Further, intervention level 3 was associated with a reduction in cases in the Pacific region, but an increase in cases in North Central States. Intervention level 1 was the only intervention not associated with any changes to COVID-19 rates across all US Census regions.

**Table 3:** Multivariate model results^*^ for case incidence and doubling time by US census region.

		**Pacific states**.		**Mountain states**		**North central states**		**South central states**		**South Atlantic states**		**Middle Atlantic & New England**	
		**Log beta**	**(95% CI)**	**Log beta**	**(95% CI)**	**Log beta**	**(95% CI)**	**Log beta**	**(95% CI)**	**Log beta**	**(95% CI)**	**Log beta**	**(95% CI)**
Case incidence model results	10-Day compliance	0.328	(0.24–0.42)^†††^	0.222	(0.15–0.29) ^†††^	−0.039	(−0.09–0.01)	0.08	(0.02–0.14)^†^	0.25	(0.19–0.31)^†††^	0.332	(0.23–0.43)^†††^
	**Duration of intervention (days)**												
	Level 1	0.016	(−0.03–0.06)	0.1	(0.02–0.18)^†^	0.013	(−0.04–0.07)	0.033	(−0.03–0.1)	0.047	(−0.01–0.1)	0.076	(0.03–0.12)^††^
	Level 2	−0.095	(−0.2–0.01)	−0.2	(−0.27–0.13)^†††^	−0.021	(−0.07–0.02)	−0.077	(−0.13–0.02)^††^	0.061	(0.02–0.1)^††^	0.16	(0.09–0.23)^†††^
	Level 3	−0.103	(−0.21–0.005)	−0.037	(−0.09–0.0143)	0.049	(0.03–0.07)^†††^	0.015	(−0.02–0.05)	0.034	(−0.01–0.08)	0.035	(−0.03–0.1)
	Level 4	−0.147	(−0.18–0.11)^†††^	−0.047	(−0.1–0.004)	0.028	(0.01–0.05)^†^	0.01	(−0.02–0.04)	0.001	(−0.04–0.04)	0.037	(−0.02–0.09)
	10-day compliance* **duration of intervention**												
	Level 1	−0.002	(−0.01–0.004)	−0.008	(−0.02–0.002)	0.009	(0.002–0.016)^††^	−0.007	(−0.01–0.001)	−0.015	(−0.02–0.008)^††^	−0.01	(−0.02–0.001)^†^
	Level 2	−0.013	(−0.03–0.003)	0.007	(0–0.014)	0.013	(0.01–0.018)^†††^	0.012	(0.01–0.019)^††^	−0.014	(−0.02–0.008)^†††^	−0.016	(−0.03–0.007)^††^
	Level 3	−0.013	(−0.02–0.001)^†^	−0.004	(−0.01–0.002)^†††^	−0.002	(−0.003–0.001)^†††^	0.001	(−0.001–0.003)	−0.006	(−0.008–0.004)^†††^	−0.012	(−0.02–0.005)^††^
	Level 4	−0.002	(−0.003–0.001)^†††^	−0.003	(−0.004–0.002)^†††^	−0.001	(−0.001–0.001)^†††^	−0.002	(−0.002–0.002)^†††^	−0.001	(−0.001–0.001)^††^	−0.004	(−0.004–0.004)^†††^
Doubling time model results	10–day compliance	−0.072	(−0.119–0.024)^††^	−0.043	(−0.07–0.014)^††^	0.126	(0.105–0.147)^†††^	−0.012	(−0.034–0.009)	0.043	(0.025–0.062)^†††^	−0.101	(−0.142–0.06)^†††^
	**Duration of intervention (days)**												
	Level 1	0.077	(0.041–0.112)^†††^	0.001	(−0.045–0.048)	0.144	(0.112–0.177)^†††^	−0.083	(−0.116–0.049)^†††^	−0.058	(−0.085–0.032)^†††^	−0.029	(−0.057–0)^†^
	Level 2	0.265	(0.197–0.333)^†††^	−0.029	(−0.11–0.052)	0.022	(−0.007–0.052)	−0.105	(−0.133–0.077)^†††^	−0.021	(−0.042–0.001)	−0.085	(−0.172–0.003)
	Level 3	0.361	(0.279–0.444)^†††^	0.116	(0.035–0.197)^††^	−0.002	(−0.022–0.019)	−0.008	(−0.027–0.011)	0.013	(−0.008–0.034)	−0.208	(−0.297–0.119)^†††^
	Level 4	0.376	(0.305–0.447)^†††^	0.129	(0.048–0.21)^††^	0.014	(−0.006–0.035)	−0.027	(−0.044–0.009)^††^	−0.024	(−0.045–0.003)^†^	−0.128	(−0.216–0.04)^††^
	10-day compliance* **duration of intervention**												
	Level 1	0.003	(0.001–0.005)^††^	−0.004	(−0.008–0.001)^†^	−0.022	(−0.024–0.02)^†††^	0.002	(0–0.004)	0.004	(0.002–0.006)^†††^	−0.001	(−0.003–0.002)
	Level 2	0.008	(0.002–0.014)^†^	0.011	(0.007–0.02)^†††^	−0.005	(−0.007–0.003)^†††^	0.01	(0.008–0.012)^†††^	−0.004	(−0.005–0.002)^†††^	0	(−0.003–0.003)
	Level 3	0.011	(0.006–0.016)^†††^	0	(0–0.001)	0	(0–0)	−0.003	(−0.003–0.002)^†††^	−0.003	(−0.004–0.003)^†††^	0.008	(0.006–0.011)^†††^
	Level 4	0.002	(0.002–0.003)^†††^	0.001	(0.001–0.001)^†††^	0	(0–0)	0.001	(0.001–0.001)^†††^	0.0002	(0–0)^†††^	0.003	(0.003–0.003)^†††^

††† *p < 0.0001*,

†† *p < 0.01*,

† *p < 0.05*.

* *Results are from 2 separate sets of negative binomial models, six per set for each Census region. Estimates are log beta incidence rates where negative values indicate reductions in the outcome (i.e., lower case rates or lower doubling time) and positive values indicate increases in the outcome. The full multivariate models are shown in Supplementary Table B1 for case incidence and Supplementary Table C1 for doubling time*.

As noted, policy compliance impacted policy effectiveness at national and Census region levels. Each unit increase in compliance was associated with a log beta decline in case rates of 0.002 and 0.001 for policy level 3 and 4, respectively (95% CI, −0.0024 to −0.0016; −0.0012 to −0.0008 respectively) at the national level. Compliance during level 4 policies was significantly associated with reduced case rates across all US Census regions (Supplementary Table B1).

#### Doubling Time

**Figure 4:**
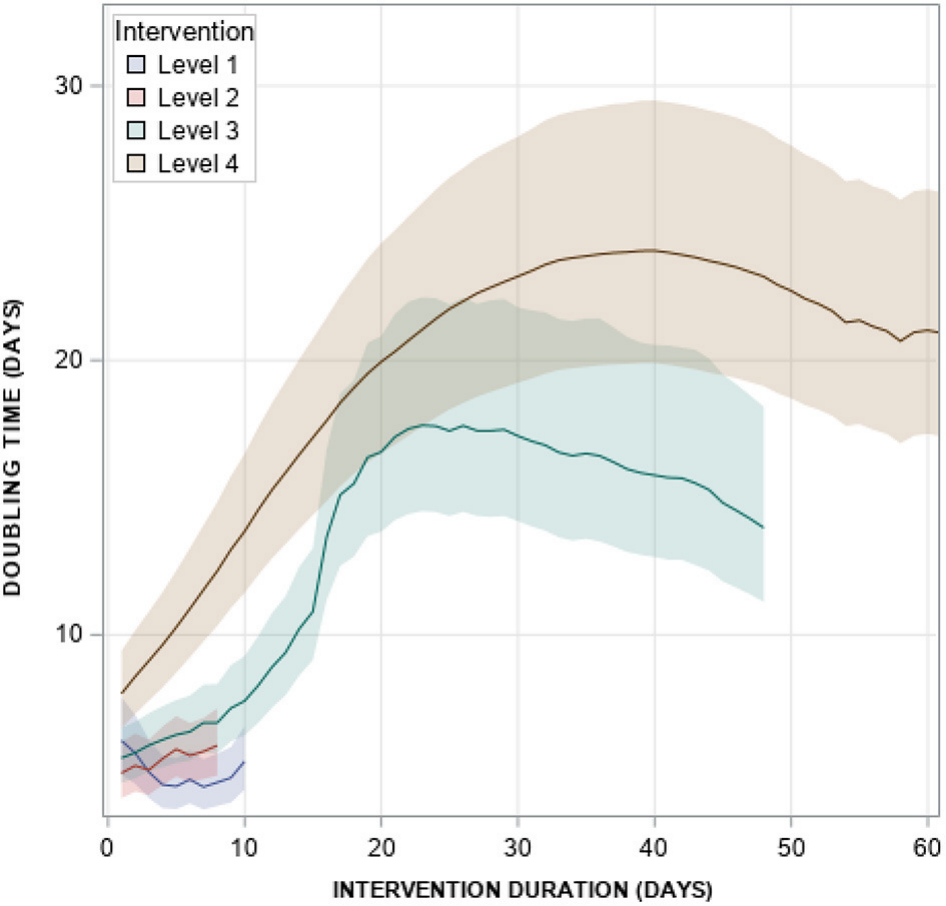
Model-predicted doubling time by policy intervention level. Model predictions are from the final Doubling Time model shown in Supplementary Table B0.

For the country as a whole, only duration of policy level 4 achieved statistical significance to increase doubling time (Table 2). Levels 2 and 3 had p-values under 0.05, but after adjusting for multiple comparisons, we cannot reject the null hypothesis of no effect; however, it is noteworthy their effect sizes were positive, indicating that the level 2 and 3 policies trended toward reduction of overall COVID-19 propagation. Each day on intervention level 4 was associated with an increase in log beta doubling time of 0.02 (95% CI: 0.01–0.03, *p* < 0.0001). When calculating the predicted doubling time from the data, level 4 policies achieve a peak 40 days after initiation with an estimated doubling time of 24 days (95% CI, 19.1–29.5) compared to 23 days after initiation of level 3 policies for an estimated doubling time of 17.6 days (95% CI, 14.5–22.3) (Figure 4). Levels 1 and 2 never achieve increased doubling times.

By US Census region, duration of level 4 policies in Pacific, Mountain and North Central states have a positive effect on doubling time, but in other regions, duration of level 4 policies reduced doubling time (Table 3, Supplementary Table C2). In addition, level 3 policy duration was significantly related to longer doubling times in Pacific and Mountain states, but shorter doubling times in Middle Atlantic and New England states. Level 1 policy duration was significantly associated with shorter doubling times in South Central, South Atlantic, Middle Atlantic and New England states, and longer doubling times in Pacific and North Central states.

Policy compliance was an important factor to explain doubling time. At the national level, higher policy compliance increased doubling time (log beta 0.036, 95% CI 0.03–0.04), yet higher compliance during policies 1 through 3 reduced doubling time. In other words, both policy level 3 and compliance increase doubling times; however, as compliance increases, the effect of policy level 3 declines. By Census region, the effect of compliance varied, increasing doubling time in North Central and South Atlantic states, but reducing doubling times in others.

#### Deaths

This analysis includes data from 73,676 COVID-19 deaths, the majority in Middle Atlantic (36%), East North Central (19%), New England (15%), South Atlantic (11%), and Pacific (7%) states. For the country as a whole, duration of policy levels 3 and 4 were both significantly associated with higher death rates (Table 2); however, the distribution of deaths indicate potential heterogeneity. Indeed, model results by Census region indicate duration of levels 1 and 2 had no effect on death rates for any Census region (Supplementary Table D1); level 3 policy duration was significantly related to lower COVID-19 death rates in North Central states, yet higher death rates in South Central, South Atlantic, Middle Atlantic, and New England states; and duration of level 4 was only related to lower death rates in Pacific states (other regions with *p*-values under 0.05 were not significant after the Holm-Bonferroni adjustment). We note that models for death rates had to be simplified with fewer covariates to allow for model convergence.

**Figure 5:**
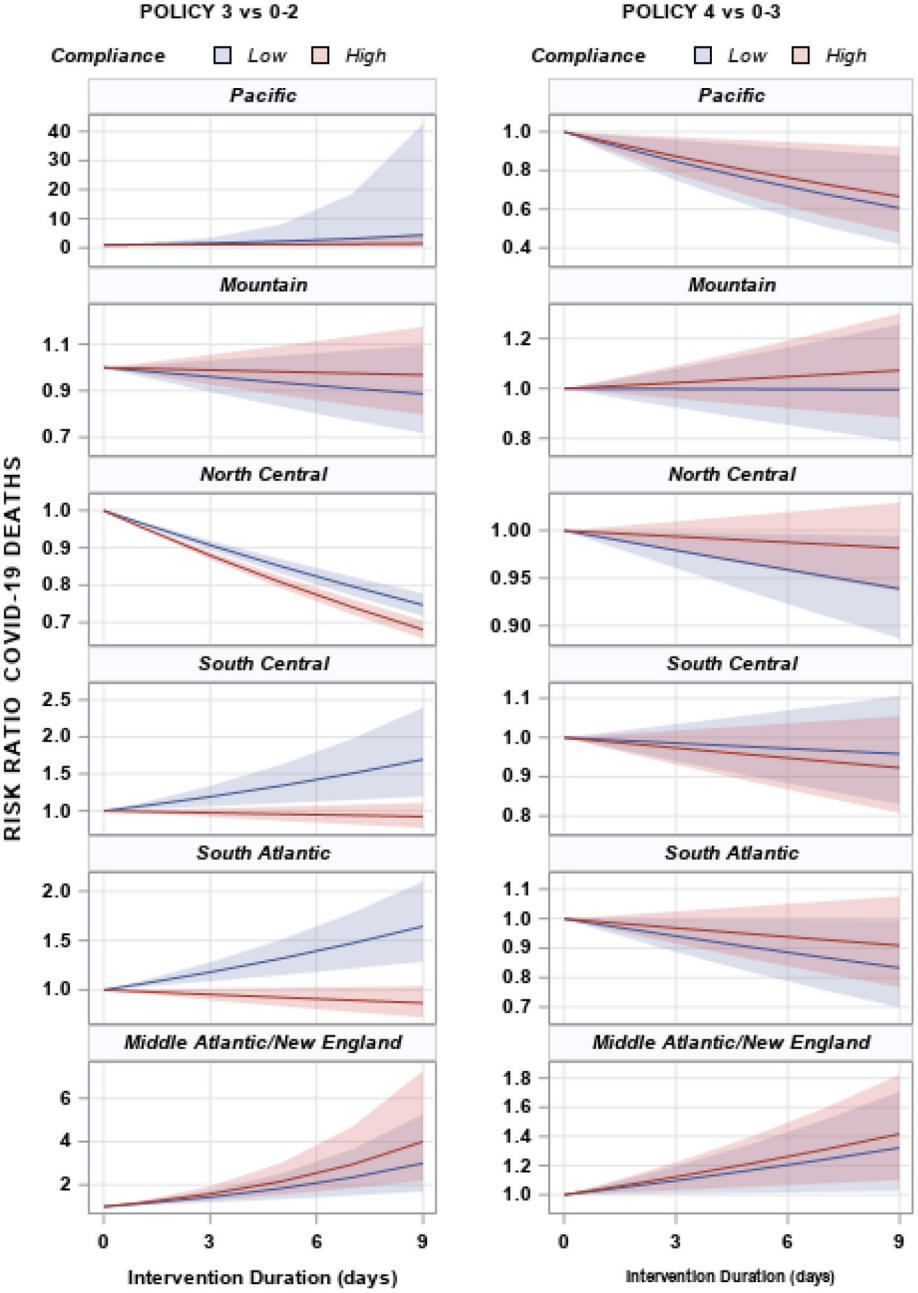
Risk ratio and 95% confidence intervals of COVID-19 deaths by duration of policy for each US census region. Shaded areas represent the 95% confidence interval for each region. US Census Regions: Pacific—CA, WA, OR, HI, AK; Mountain—MT, WY, ID, NV, UT, CO, AZ, NM; North Central—ND, SD, NE, KS, MN, IA, MO, WI, IL, MI, IN, OH; South Central—TX, OK, AR, LA, KY, TN, MS, AL; South Atlantic—FL, GA, SC, NC, VA, WV, DC, MD, DE; Middle Atlantic—NY, PA, NJ; New England—ME, CT, NH, MA, CT, RI.

The effect of policy duration on death rates is sensitive to measures of compliance. This is particularly true for level 3 policies as seen in Figure 5, where high levels of compliance resulted in significantly lower death rates that low levels of compliance in North Central, South Central, and South Atlantic states. Policy compliance did not influence the effectiveness of other policies.

### Social and Environmental Disparities

As noted in Methods, we conducted a secondary evaluation of social and environmental factors associated with COVID-19 cases and deaths, as well as factors related to policy compliance. These variables include county-level measures of: poverty, unemployment and income; average level of educational attainment; socio-demographic characteristics (population density, minority population, %Black, %Hispanic); and hydro-meteorological characteristics. Model results related to policy compliance are reported in Table 4.

**Table 4:** Socioeconomic characteristics associated with policy compliance (changes in mobility) during the first 15 and 30 days of March 2020.

		**Compliance change**		**Compliance change**	
		**March 1 to March 15**		**March 1 to March 30**	
		**Beta (SE)**	**Standardized beta (SE)**	**Beta (SE)**	**Standardized beta (SE)**
Intercept		0.225 (0.547)	2.32 (0.253)^†††^	19.614 (0.982)^†††^	25.17 (0.338)^†††^
% Of county population:	With Bachelor degree	0.056 (0.008)^†††^	28.68 (3.899)^†††^	0.097 (0.015)^†††^	49.44 (7.557)^†††^
	Hispanic	0.009 (0.006)	6.66 (4.344)	0.028 (0.011)^††^	21.50 (8.278)^††^
	Black	−0.012 (0.006)^†^	−9.97 (4.916)^†^	−0.015 (0.012)	−11.99 (9.473)
	Living in Poverty	0.024 (0.013)	7.94 (4.462)	−0.114 (0.026)^†††^	−38.35 (8.656)^†††^
Net migration rate		−0.006 (0.006)	−3.54 (3.505)	0.057 (0.011)^†††^	35.98 (6.788)^†††^
2018 County population density, 1,000 people/km^2^		−0.112 (0.098)	−3.44 (3.021)	0.12 (0.191)	3.69 (5.877)
Rural-urban continuum code (Ref = 9-Rural <2,500 people, not adjacent to metro)	1—Metro >1 million people	1.025 (0.251)^††^	19.35 (4.74)^††^	5.431 (0.488)^†††^	102.48 (9.198)^†††^
	2—Metro, 250 K-−1 million people	1.869 (0.233)^†††^	33.83 (4.226)^†††^	6.13 (0.454)^†††^	110.96 (8.214)^†††^
	3—Metro, <250 K people	2.122 (0.226)^†††^	37.32 (3.97)^†††^	5.769 (0.439)^†††^	101.47 (7.721)^†††^
	4—Non-metro, >20 K people, adjacent to metro area	2.47 (0.258)^†††^	34.77 (3.635)^†††^	6.548 (0.502)^†††^	92.19 (7.065)^†††^
	5—Non-metro, >20 K people, not adjacent to metro area	2.429 (0.338)^†††^	22.58 (3.145)^†††^	6.771 (0.659)^†††^	62.95 (6.128)^†††^
	6—Non-metro, 2,500–19,999 people, adjacent to metro area	1.542 (0.197)^†††^	33.63 (4.298)^†††^	5.425 (0.383)^†††^	118.29 (8.357)^†††^
	7—Non-metro, 2,500–19,999, not adjacent to metro area	1.573 (0.203)^†††^	30.01 (3.878)^†††^	5.248 (0.396)^†††^	100.14 (7.55)^†††^
	8—Rural, <2,500 people, adjacent to metro	0.889 (0.244)^††^	12.68 (3.485)^††^	2.058 (0.476)^†††^	29.36 (6.789)^†††^
Minimum temperature		−0.08 (0.017)^†††^	−37.14 (8.103)^†††^	0.012 (0.031)	5.68 (14.249)
Solar radiation		−0.003 (0.002)	−6.84 (4.315)	0.002 (0.004)	3.56 (8.194)

††† *p < 0.0001*,

†† *p < 0.01*,

† *p < 0 .05*.

*Results are from two separate models of compliance change over time, adjusting for within state county correlation. The interpretation of the standardized beta is a one standard deviation unit change in a covariate being associated with a change in compliance*.

#### Poverty

Poverty and income data used in this study are from the US Census Bureau's Small Area Income and Poverty Estimate (SAIPE). At the national level, as expected, higher levels of poverty are associated with shorter doubling times and higher death rates, yet, counterintuitively, lower case rates. When evaluating poverty effects by Census region, poverty was not associated with doubling times in any region, but was positively associated with case rates in Mountain states and inversely related to cases in all other regions. In contrast, elevated poverty was consistently associated with elevated COVID-19 death rates in all regions. Although poverty was not associated with 15-day change in compliance, a county's poverty level was the most important factor explaining low compliance with any policy after 30 days.

#### Education

Education data are 5-year averages estimated by the American Community Survey. At the national level, the percent of the county with at least a Bachelor's degree was not associated with case rates, but was associated with increased doubling times. By Census region, higher education was consistently associated with increased doubling time, yet variability existed when analyzing case rates with education associated with higher case rates in Mountain, Middle Atlantic, and New England states, but lower cases in South Central states. Higher education was also consistently associated with increased policy compliance, for both the 15 and 30 day changes in compliance.

#### Socio-Demographic

Social and demographic data are county racial, ethnic and total population characteristics: %Black, %Hispanic, population density, and the USDA Urban-Rural Classification. None of these factors were identified as related to death rates and population density was not evaluated in case rate models as population size is used as an offset in the model. At the national level, %Black and %Hispanic populations were associated with elevated case rates, which was consistent across Census regions. Case rates were also consistently higher in metropolitan areas as well as non-metropolitan areas with more than 20,000 people compared to smaller counties (i.e., non-metro areas under 20,000 people and rural counties). By Census region, %Black and %Hispanic were both associated with elevated cases in North Central, South Atlantic, Middle Atlantic, and New England states. %Black was also associated with elevated cases in South Central states while %Hispanic was associated with elevated cases in Pacific states. Case rates by urban-rural classification varied considerably by Census region: Pacific and South Atlantic states had similar patterns with smaller counties having higher rates that larger counties; Middle Atlantic and New England states had the opposite patterns (large counties had higher rates compared to smaller counties); and the other Census regions had mixed patterns. Counties with the highest policy compliance levels tended to have larger %Hispanic, lower %Black populations, and be in counties with larger population sizes (i.e., non-metropolitan counties with >20 K people and larger).

#### Climate

Hydrometeorological data were used to define 10-day temporal lags of minimum air temperature, humidity, and shortwave radiation. The relationship between climate parameters and COVID-19 was variable, and these relationships often differed between case doubling time and deaths (Supplementary Figures D1, D2). Solar radiation, for example, which is frequently invoked as a negative forcing on COVID-19 transmission on account of its relationship with UV radiation intensity (50, 51), is associated with increased case doubling time (i.e., decreased transmission) in coastal regions but with decreased doubling time in the North Central and South Atlantic regions. For deaths, most northern census regions (Middle Atlantic, New England, North Central, Mountain) had tend toward decreased deaths with increased solar radiation, but southern regions had mixed relationships.

Increases in specific humidity and minimum temperature were associated with decreased doubling time and increased deaths for the county as a whole (specific humidity: log beta −0.006, 95% CI −0.009 to −0.003 for doubling time, 0.015, 95% CI 0.003–0.027 for deaths; minimum temperature: log beta −0.005, 95% CI −0.006 to −0.003 for doubling time, log beta 0.015, 95% CI 0.0075-0.0225 for deaths). In the South Central, South Atlantic and Middle Atlantic/New England Census regions, minimum temperature and specific humidity exhibited the same inverse relationship with doubling time (i.e., higher values, shorter doubling times) and positive relationship with death rates (higher values, higher death rates). Other regions had contrasting relationships. For example, specific humidity was significantly associated with lower doubling times in Pacific states, but higher doubling times in North Central; however, specific humidity had a (non-significant) downward trend with death rates in those two regions.

Regarding policy compliance, after 30 days, no climate factors were associated with compliance.

## Discussion

This study evaluates the effectiveness of four non-pharmaceutical intervention categories on COVID-19 case rates, doubling time, and deaths at the county level in the US, and the heterogeneity that exists across Census regions. We find that during the first wave of COVID-19, the most restrictive NPI policies (level 4) were the most effective at reducing case rates and increasing doubling time of COVID-19 compared to any other policy level. In addition, we observed that higher levels of policy compliance, as measured by changes in county-level mobility from 2019 to 2020, resulted in larger reductions in cases but lower increases in doubling time for these restrictive NPI. Analysis of NPI effectiveness across Census regions revealed strong variation across regions and within states. Level 4 policies were associated with reduced case incidence only in Pacific states and increased doubling time only in Mountain states, yet associated with higher case rates in North Central States and lower doubling times in multiple regions, noting in particular that the effect sizes for doubling time in Mountain vs. Middle Atlantic/New England states were exactly the same value but in opposite directions.

Surprisingly, duration of level 4 policies was associated with higher rates of death at the national level, but when analyzed separately by Census region, associated with lower rates of death in Pacific, North Central, and South Atlantic states (also trended to lower rates in Mountain and South Central regions, but was not statistically significant). Given than Middle Atlantic and New England states comprised 51% of all deaths reported during the first wave, the relationship in these two regions likely dominated the national trend. The initiation of level 4 policies was slowest in these regions, allowing for both case and mortality momentum that resulted in the positive association observed. It is clear from Supplementary Table D1 that level 4 policies not only reduced death rates in other regions, but higher compliance may complement policy effectiveness.

Why do NPIs exhibit such variation in effects? As alluded to above, policy compliance likely plays a key role, which we measured as the change in mobility over a 10-day period in 2019 vs. 2020. It is not surprising that we find compliance to be associated with enhanced policy effectiveness and reduced COVID-19 burden, i.e., higher compliance was significantly related to lower case rates and higher doubling times for each day on level 4 policies in all Census regions, as well as lower death rates in most regions during both level 3 and level 4 policies. However, we found considerable variation in compliance both within Census regions and within states (Figure 2, Supplementary Figure A1) indicating that adherence to policies has strong community and census-level mediating factors. This is consistent with our findings that that compliance varies by levels of poverty, educational level, racial/ethnic composition, and rurality. Recent studies corroborate these findings and also highlight the importance of perceived risk, occupation, and local social pressures (52–55). Importantly, we find poverty levels to be the most important factor in explaining compliance, which was also found in other studies (52, 56). This relationship between poverty, policy compliance, and COVID-19 risk is tied to the overall literature of population vulnerability and the burdens of low-income households to maintain food security and financial stability during a time when businesses and employment opportunities were closing (57).

Overall, the results of this study are consistent with findings from Wuhan (23) and Europe (58) that reported significant declines in the effective reproductive number following implementation of NPIs that included quarantine, travel restriction, shelter-in-place, school and business closures, and social distancing. Results are also consistent with studies in the US highlighting the effect of shelter-in-place (5, 59) and closure of schools, restaurants and businesses (26–28). However, in contrast to these studies, we combine multiple NPIs into an ordered grouping of policies that tended to be implemented simultaneously, thereby avoiding the potential biases in assigning attributable risk reduction to individual policies when their roll-out occurs concurrently with others. For example, 51% of counties had school and restaurant closures occur <2 days apart (level 2 NPI), and 55% initiated restrictions on mass gatherings and non-essential business closures within 2 days (level 3 NPI). It is very difficult, statistically, to measure independent policy effects in an observational study when those policies are implemented in such close temporal proximity. The stepped-wedge approach, although not designed for observational data, is a novel attempt to control for these overlapping periods.

The stepped wedge approach was only applied to the doubling time models, primary due to difficulties in achieving model convergence for case and death rate models. For doubling time, we produced predicted estimates for each NPI adjusting for all covariates entered into the model (Figure 4, Supplementary Figure C1). These predicted doubling times adjust for all the observed heterogeneity across counties, demonstrating that policy impact is likely not continuous in reality, but achieves a peak after a certain amount of time. Overall, we report level 4 achieving peak doubling time after 40 days and 23 days for level 3. This peaking is likely associated with reduced perceptions of local risk and reduced compliance even when policies are still in effect. For Census regions, they all appear to reach a peak doubling time for level 4 policies with the exception of Middle Atlantic and East North Central, which tended to have less heterogeneity in policy compliance compared to other regions as indicated in Figure 2. This plateauing of intervention effects remains an important area of research, which is tied to issues related to adherence, transportation, and racial and economic disparities. The timing of policy implementation may also have introduced heterogeneity across regions. We observed that states experiencing early cases (Pacific, New England, and Mid-Atlantic) had significantly longer gap times between case detection and NPI initiation than states experiencing their first COVID-19 case later. This difference in policy initiation time is likely due to non-coastal US Regions learning from experience of coastal regions to implement NPIs more quickly. Unfortunately, early response during the first wave, which proved effective, may have given policy-makers false confidence as 12 states reported case spikes on June 23 and 7 states reported highs for hospitalization (https://covidtracking.com/)—all but one of these states began removing social distancing protections by May 11 and all but four are located in Mountain, North and South Central regions.

In addition to evaluating policy effects, this study reported the effects of temperature, specific humidity, and solar radiation related to COVID-19. These mixed results highlight a number of important points on climate predictors of COVID-19: (1) the regional heterogeneity of the relationship between climate parameters and SARS-CoV-2 is consistent with the variability observed in studies of MERS-CoV infection and climate (60–63); (2) climate analyses are sensitive to choice of response variable and analysis period, and may not provide stable results at the stage of epidemic data collection considered in this study—for example, our results tend to run in the opposite direction of those reported by Ma et al. (64), but the studies use different response variables (*R*_t_ vs. doubling rate), as well as different periods of analysis, ranges of climate variability, and statistical methods; (3) there is substantial inter-regional variability in climate sensitivities, such that large scale analyses are not necessarily representative of regional climate influence; and (4) it is difficult to isolate climate effects from those of other predictors, and analyses that examine climate variables without adequate control for policy and other factors are prone to spurious climate associations. We recommend further research be conducted at different spatial scales (community, census tract, etc.) to better characterize the climate-COVID-19 relationship.

### Limitations

There are several limitations to note in our study. First, the analysis does not have an accurate representation of the availability of testing (or the number of tests administered) at the county level for the time series. As this availability changed over time for all counties in the US, we cannot accurately characterize the population at risk for COVID-19 case detection. However, we are more confident in COVID-related deaths reported. Second, our measure of compliance is based on reduced mobility in countries, but not a direct measure of adherence. In some states, policy levels 3 and 4 were viewed as an affront to civil liberties. While the use of mobility data has been shown to be an appropriate proxy for compliance, studies focusing on smaller geographic units may be able to obtain more accurate data on compliance to measure NPI “dose.” Third, we do not look at individual interventions. However, this was a choice as the sets of NPIs are considered a more appropriate response than single interventions, which have never been employed historically without others. Finally, we would ideally be applying these methods to a randomized design, which is impossible for COVID-19. Thus, our inferences are drawn from observational data regardless of our SW-CRT analytical framework.

## Conclusion

The most aggressive NPIs (shelter-in-place, public mask requirements, and travel restrictions) were the only policies that are consistently associated with a reduction in COVID-19 cases and doubling times in the US between January 2020 and the phased re-opening of states. However, when analyzing by Census region, considerable variation of NPI effectiveness is observed, likely due to variations in policy adherence. Socio-environmental factors, including poverty, racial/ethnic status and educational levels, contribute to heterogeneity of COVID-19 propagation, NPI adherence and NPI effectiveness. These results may inform public health policy as states continue to manage the ongoing pandemic.

## Data Availability Statement

The raw data supporting the conclusions of this article will be made available by the authors, without undue reservation.

## Author Contributions

WP and ST conceived the study. WP, ST, BZ, PL, and CW contributed to the design of the study. WP, RD, and BZ organized the data. WP, DF, and G-VI contributed to analysis. WP wrote the first draft of the manuscript. All authors contributed to manuscript revision, read, and approved the submitted version.

## Conflict of Interest

The authors declare that the research was conducted in the absence of any commercial or financial relationships that could be construed as a potential conflict of interest.

## Publisher's Note

All claims expressed in this article are solely those of the authors and do not necessarily represent those of their affiliated organizations, or those of the publisher, the editors and the reviewers. Any product that may be evaluated in this article, or claim that may be made by its manufacturer, is not guaranteed or endorsed by the publisher.
