# Supplementary material for: Heterogeneity in the Effectiveness of Non-pharmaceutical Interventions During the First SARS-CoV2 Wave in the United States
Source: Front Public Health. 2021 Nov 29;9:754696. doi: 10.3389/fpubh.2021.754696 (PMC8666663; doi:10.3389/fpubh.2021.754696)
Supplement: Supplementary file 1 [file Data_Sheet_1.PDF]

SUPPLEMENTAL TABLES AND FIGURES

Supplemental Table A1. Average duration of policies by state

Supplemental Figure A1. Compliance of Policy Interventions for US Counties by US State.
Each dot represents a county and each county has a daily time series of compliance. The red line is a fitted penalized b-spline.

Supplemental Table B0. Final Multivariate Model Results for COVID-19 Case Incidence Rate and Doubling Time (log scale, All US Census Regions)

Supplemental Table B1. Multivariate models evaluating new case detection by US Census Region

Supplemental Figure B1. Intervention Effects on COVID-19 Cases by US Census Region and Policy Compliance
Results are from the 6 models shown in Supplemental Table B1.

Supplemental Table C1. Multivariate Models for Doubling Time by US Census Region
Results are from 6 negative binomial models, one for each US Census Region. It includes Period (day of time series) where period 1 is equivalent to January 22, 2020. Period is included in this model as a categorical variable, consistent with the Stepped Wedge model specification.

Supplemental Figure C1. Predicted Doubling Time from observed data by duration of intervention for each Census Region with 95% confidence intervals.
Estimates are mean policy intervention effects for each US region based on model results in Supplemental Table C1 and the observed data from each region. Note that increases in doubling time do not indicate that the policy effect is significant; rather, there are several factors influencing predicted doubling time.

Supplemental Table D1. Multivariate Model Results for COVID-19 Death Rates
Results from this table are from three county-level models for each US Census Region (one for each intervention level). Period refers to the study day (day 1=January 22, 2020); Policy Level is an indicator for whether a policy is in place or not; and Policy Duration is days the policy is in effect.

Supplemental Figure D1. Climate Parameter Effects on COVID-19 Doubling Time by US Census Region
Results are from 3 separate doubling time models, one for each lagged climate variable, adjusting for period, rural-urban classification, % of county with a bachelor degree or higher, US Census Region, Intervention and duration of intervention. Climate is included as a fixed effect plus an interaction with Census Region. The overall effects of specific humidity (log beta -0.046, 95% CI -0.07- -0.027) and solar radiation (log beta 0.0004, 95% CI 0.0001-0.0008) are significant. The climate effect and 95% CI for each region are estimated from predicted log-beta values from the overall effect plus Census Region interaction.

Supplemental Figure D2. Climate Parameter Effects on COVID-19 Deaths by US Census Region
Results are from 3 separate death rate models, one for each lagged climate variable, adjusting for period, rural-urban classification, Census Region, Intervention level and duration. Temperature and solar radiation models included county %Bachelor degree; the specific humidity model includes county %Black and %Hispanic. The overall effects of minimum temperature (log-beta 0.04511, 95% CI: 0.025-0.065) and specific humidity (log beta 0.0524, 95% CI: 0.005-0.1) are significant. The climate effects and 95% CI for each region are estimated from the predicted log-beta values from the overall effect plus the Census Region interaction.

Supplemental Table A1. Average duration of policies by state

State	Level 1 (95% CI)		Level 2 (95% CI)		Level 3 (95% CI)		Level 4 (95% CI)	
**AK**	5.0	(4.2-5.9)	3.0	(2.4-3.7)	8.1	(7.2-9.2)	47.9	(45.4-50.4)
**AL**	5.8	(5.2-6.4)	0.1	(0-0.2)	16.0	(15.1-17)	43.2	(41.6-44.8)
**AR**	5.7	(5.2-6.3)	2.3	(2-2.6)	0.0		59.0	(57.3-60.8)
**AZ**	4.7	(3.7-5.9)	0.0		15.0	(13.2-17.1)	44.0	(40.8-47.5)
**CA**	11.0	(10.2-11.9)	3.3	(2.8-3.8)	0.2	(0.1-0.3)	72.6	(70.4-74.8)
**CO**	4.0	(3.5-4.5)	4.0	(3.5-4.5)	7.9	(7.2-8.6)	42.1	(40.6-43.7)
**CT**	3.0	(2-4.5)	10.0	(8-12.4)	0.0		64.0	(58.7-69.8)
**DC**	3.0	(1-9.3)	8.0	(4-16)	8.0	(4-16)	59.0	(45.7-76.1)
**DE**	4.0	(2.3-7)	8.0	(5.4-11.9)	0.0		67.0	(58.3-76.9)
**FL**	8.0	(7.4-8.7)	3.0	(2.6-3.4)	11.8	(11-12.7)	39.2	(37.7-40.7)
**GA**	5.0	(4.7-5.4)	1.0	(0.9-1.2)	14.2	(13.6-14.8)	30.8	(29.9-31.7)
**HI**	13.0	(10.2-16.6)	0.0		0.0		74.0	(66.8-81.9)
**IA**	7.0	(6.5-7.5)	4.0	(3.6-4.4)	55.0	(53.6-56.5)	0.0	
**ID**	5.0	(4.4-5.7)	0.0		0.0		65.0	(62.7-67.4)
**IL**	8.0	(7.5-8.6)	3.0	(2.7-3.4)	1.0	(0.8-1.2)	70.0	(68.4-71.6)
**IN**	6.0	(5.5-6.5)	4.0	(3.6-4.4)	7.9	(7.4-8.5)	47.1	(45.7-48.5)
**KS**	6.0	(5.5-6.5)	0.0		11.2	(10.6-11.9)	41.8	(40.5-43)
**KY**	4.0	(3.7-4.4)	5.0	(4.6-5.4)	11.0	(10.4-11.6)	63.0	(61.6-64.4)
**LA**	1.0	(0.8-1.3)	6.0	(5.4-6.6)	5.0	(4.5-5.6)	59.0	(57.1-60.9)
**MA**	5.0	(4-6.3)	1.0	(0.6-1.7)	8.0	(6.6-9.6)	67.0	(62.8-71.4)
**MD**	0.0		11.0	(9.8-12.4)	14.0	(12.6-15.6)	52.0	(49.2-55)
**ME**	0.0		3.0	(2.3-4)	14.0	(12.3-16)	59.0	(55.4-62.9)
**MI**	6.0	(5.5-6.6)	4.0	(3.6-4.5)	4.0	(3.6-4.5)	67.0	(65.3-68.8)
**MN**	6.0	(5.5-6.5)	2.0	(1.7-2.3)	8.0	(7.4-8.6)	58.0	(56.4-59.6)
**MO**	6.0	(5.6-6.4)	4.0	(3.6-4.3)	11.6	(11-12.3)	36.4	(35.3-37.6)
**MS**	5.0	(4.5-5.5)	0.0		15.0	(14.1-15.8)	40.0	(38.7-41.4)
**MT**	3.0	(2.6-3.5)	13.0	(12.1-14)	0.0		43.0	(41.3-44.8)
**NC**	6.0	(5.5-6.5)	4.0	(3.6-4.4)	9.5	(9-10.2)	45.5	(44.2-46.8)
**ND**	3.0	(2.6-3.5)	4.0	(3.5-4.6)	48.0	(46.2-49.9)	0.0	
**NE**	10.0	(9.3-10.6)	8.5	(7.9-9.1)	39.5	(38.3-40.8)	0.0	
**NH**	0.0		2.0	(1.3-3.1)	10.0	(8.2-12.2)	51.0	(46.8-55.6)
**NJ**	7.0	(6-8.2)	2.9	(2.2-3.7)	2.1	(1.6-2.9)	70.0	(66.5-73.7)
**NM**	5.0	(4.3-5.8)	3.0	(2.5-3.7)	5.0	(4.3-5.8)	59.0	(56.4-61.7)
**NV**	4.0	(3.2-5.1)	1.0	(0.6-1.6)	15.0	(13.3-17)	44.0	(41-47.3)
**NY**	9.0	(8.3-9.8)	4.0	(3.5-4.5)	2.0	(1.7-2.4)	69.0	(67-71.1)
**OH**	4.0	(3.6-4.4)	5.0	(4.6-5.5)	4.0	(3.6-4.4)	57.0	(55.4-58.6)
**OK**	2.0	(1.7-2.3)	8.0	(7.4-8.7)	7.0	(6.4-7.6)	36.0	(34.7-37.4)
**OR**	8.0	(7.1-9)	1.0	(0.7-1.4)	6.0	(5.3-6.9)	59.0	(56.5-61.6)
**PA**	10.0	(9.3-10.8)	1.0	(0.8-1.3)	0.0	(0-0.1)	58.0	(56.2-59.9)
**RI**	7.0	(5-9.7)	1.0	(0.4-2.4)	11.0	(8.4-14.3)	63.0	(56.4-70.4)
**SC**	3.0	(2.5-3.5)	7.0	(6.3-7.8)	14.8	(13.7-15.9)	33.2	(31.6-34.9)
**SD**	3.0	(2.6-3.4)	7.0	(6.4-7.7)	42.0	(40.5-43.6)	0.0	
**TN**	7.9	(7.4-8.5)	3.1	(2.7-3.4)	8.8	(8.2-9.4)	36.2	(35.1-37.5)
**TX**	6.0	(5.7-6.3)	1.0	(0.9-1.1)	10.9	(10.5-11.3)	37.1	(36.4-37.9)
**UT**	10.0	(8.9-11.2)	5.0	(4.2-5.9)	6.0	(5.2-7)	41.0	(38.7-43.4)
**VA**	4.0	(3.7-4.4)	8.0	(7.5-8.5)	6.0	(5.6-6.4)	52.0	(50.8-53.2)
**VT**	4.0	(3.1-5.2)	3.0	(2.2-4.1)	5.0	(4-6.3)	60.0	(56.1-64.2)
**WA**	16.6	(15.4-17.9)	2.4	(1.9-2.9)	4.0	(3.4-4.7)	68.0	(65.5-70.7)
**WI**	5.0	(4.5-5.5)	0.0		8.0	(7.4-8.7)	62.0	(60.2-63.8)
**WV**	0.0		7.0	(6.3-7.7)	1.0	(0.8-1.3)	47.0	(45.2-48.8)
**WY**	3.0	(2.4-3.8)	4.0	(3.3-4.9)	46.1	(43.4-49)	1.9	(1.4-2.5)

Supplemental Figure A1. Compliance of Policy Interventions for US Counties by US State. Each dot represents a county and each county has a daily time series of compliance. The red line is a fitted penalized b-spline.

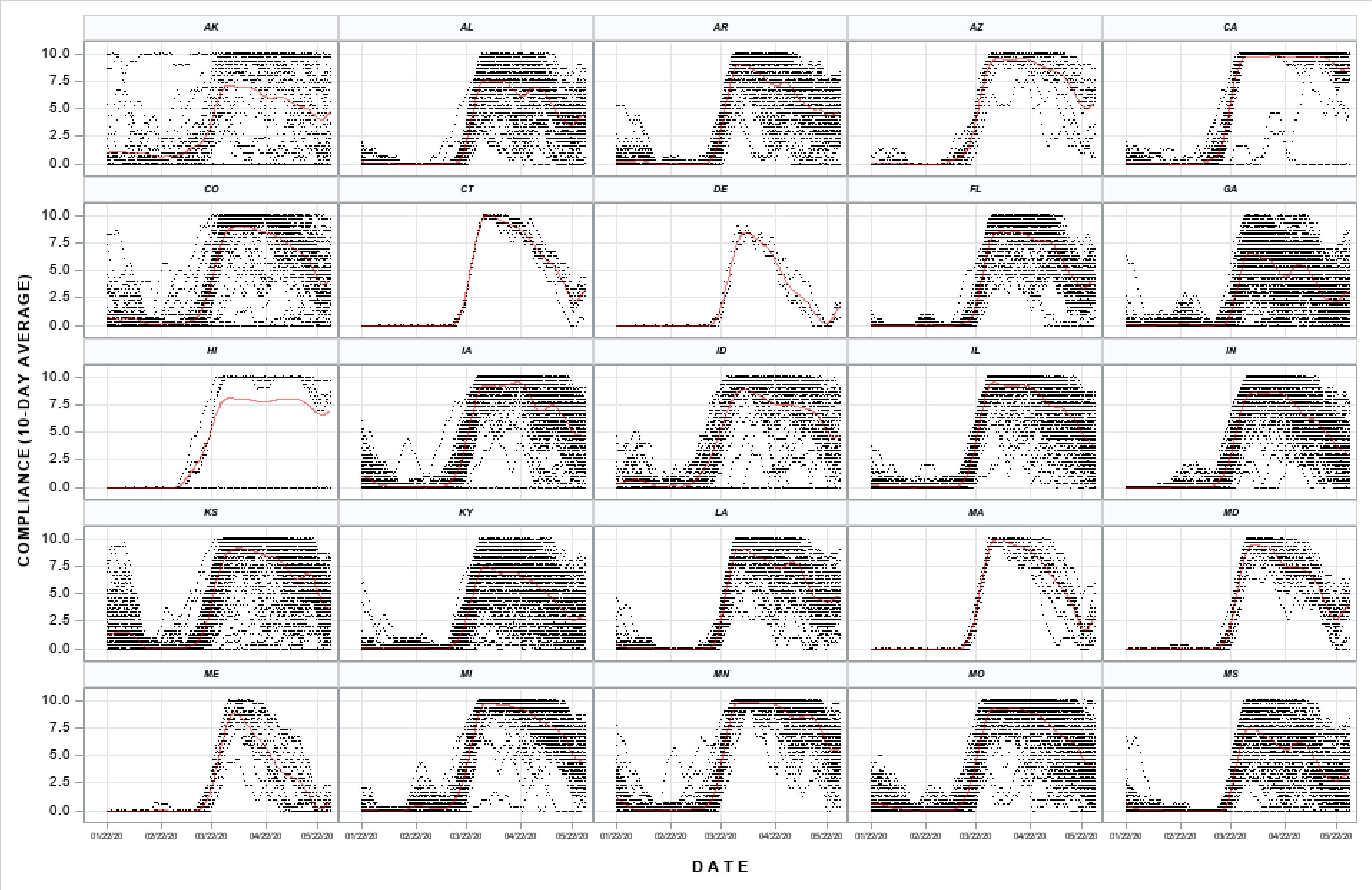

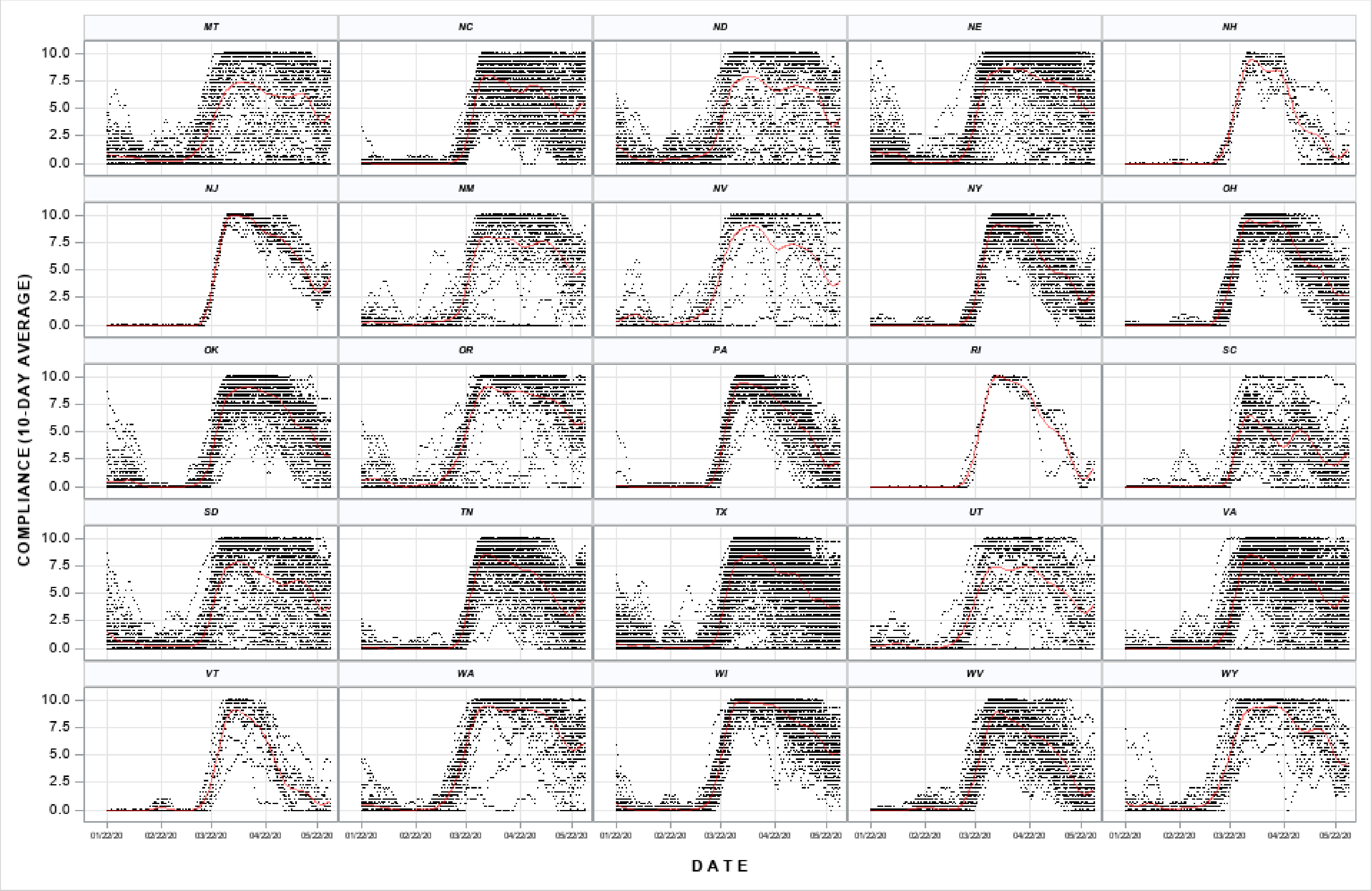
MT
NC
ND
NE
NH
NJ
NM
NV
NY
OH
OK
OR
PA
RI
SC
SD
TN
TX
UT
VA
VT
WA
WI
WV
WY
10.0
7.5
5.0
2.5
0.0
COMPLIANCE (10-DAY AVERAGE)
01/22/20
02/22/20
03/22/20
04/22/20
05/22/20
DATE

Supplemental Table B0. Final Multivariate Model Results for COVID-19 Case Detection Rate and Doubling Time (log scale, All US Census Regions)

Variable		Case Rate Model		Doubling Time Model		Death Rate Model[1]	
		Log Beta	(95% CI)	Log Beta	(95% CI)	Log Beta	(95% CI)
**Intercept**		-17.44	(-18.04--16.84) †††	0.719	(-0.09-1.52)	-4.169	(-5.32--3.02) †††
**Rural-Urban Continuum Code (Ref=9-Rural <2500 people, not adjacent to metro)**	1 - Metro >1 million people	0.213	(-0.01-0.44)	0.464	(0.35-0.58) †††		
	2 - Metro, 250K - 1 million people	-0.05	(-0.26-0.16)	0.529	(0.42-0.63) †††		
	3 - Metro, <250K people	0.205	(-0.01-0.42)	0.491	(0.39-0.6) †††		
	4 - Non-metro, >20K people, adjacent to metro area	-0.06	(-0.29-0.17)	0.553	(0.44-0.67) †††		
	5 -Non-metro, >20K people, not adjacent to metro area	0.005	(-0.3-0.31)	0.661	(0.51-0.81) †††		
	6 -Non-metro, 2500-19,999 people, adjacent to metro area	0.14	(-0.05-0.33)	0.391	(0.3-0.49) †††		
	7 -Non-metro, 2500-19,999, not adjacent to metro area;	-0.085	(-0.29-0.12)	0.299	(0.2-0.4) †††		
	8 - Rural, <2500 people, adjacent to metro	-0.001	(-0.25-0.25)	0.21	(0.09-0.33) ††		
**Percent of County Population:**	… with Bachelors degree (or higher)	-0.001	(-0.01-0.01)	0.008	(0-0.01) †††		
	… Living in poverty	-0.024	(-0.03--0.01) †††	-0.006	(-0.01-0) †	0.018	(0.01-0.02) †††
	… Hispanic	0.019	(0.01-0.02) †††	-0.002	(0-0) †		
	… Black	0.037	(0.03-0.04) †††	0.006	(0-0.01) †††		
**County Net Migration Rate, 2018**		-0.004	(-0.01-0)	0.006	(0.003-0.01) †††		
**Compliance (reduced travel), average past 10 days**		0.109	(0.09-0.13) †††	0.036	(0.03-0.04) †††	0.016	(-0.02-0.05)
**Population Density**		n/a		-0.022	(-0.06-0.02)		
**Policy Level (Ref=0 - none)**	1 – State of Emergency	0.764	(0.56-0.97) †††	0.059	(-0.03-0.15)	2.371	(1.18-3.57) ††
	2 – School, Restaurant, Bar closures, Restrict nursing home access,	1.334	(1.1-1.57) †††	0.082	(-0.03-0.19)	2.999	(1.8-4.2) †††
	3 – Non-essential business, mass gatherings, suspend non-violent arrests, evictions, procedures	1.47	(1.23-1.71) †††	0.072	(-0.04-0.19)	3.187	(2-4.38) †††
	4 – Shelter in place, travel restriction, pubic mask requirements	1.665	(1.43-1.9) †††	0.143	(0.03-0.26) †	3.539	(2.35-4.72) †††
**State Census Group (REF=9, New England)**	1 – Pacific	-0.998	(-1.39--0.6) †††	0.066	(-0.13-0.26)	-0.247	(-0.49--0.01) †
	2 – Mountain	-0.321	(-0.66-0.02)	-0.144	(-0.32-0.03)	-0.172	(-0.35-0)
	3 – West North Central	-0.168	(-0.49-0.15)	0.133	(-0.03-0.3)	0.24	(0.12-0.36) †††
	4 – East North Central	0.012	(-0.31-0.33)	-0.162	(-0.33-0)		
	5 – West South Central	-0.54	(-0.86--0.22) ††	-0.093	(-0.26-0.07)		
	6 – East South Central	-0.094	(-0.42-0.23)	-0.022	(-0.19-0.15)		
	7 – South Atlantic	-0.176	(-0.49-0.14)	-0.121	(-0.28-0.04)	0.277	(0.16-0.39) †††
	8 – Middle Atlantic	-0.154	(-0.52-0.21)	-0.069	(-0.26-0.12)	0.397	(0.24-0.55) †††
**Duration of Policy, days**	Level 1	0.042	(0.02-0.06) ††	0.004	(-0.01-0.01)	-0.018	(-0.05-0.01)
	Level 2	-0.01	(-0.03-0.01)	0.012	(0-0.02) †	0.028	(0-0.05) †
	Level 3	-0.009	(-0.02-0)	0.011	(0-0.02) †	0.062	(0.04-0.08) †††
	Level 4	-0.028	(-0.04--0.02) †††	0.02	(0.01-0.03) †††	0.056	(0.04-0.07) †††
**INTERACTION of Compliance * Policy Duration (Ref=0)**	Level 1	-0.001	(-0.003-0.001)	-0.002	(-0.003--0.001) †††	-0.0002	(-0.003-0.002)
	Level 2	0.0003	(-0.002-0.003)	-0.002	(-0.003--0.001) †††	0.0004	(-0.002-0.003)
	Level 3	-0.002	(-0.002--0.002) †††	-0.001	(-0.001--0.001) †††	-0.001	(-0.002-0)
	Level 4	-0.001	(-0.001--0.001) †††	-0.0001	(0-0)	0.0004	(0-0.001) †

†††p<0.0001, †† p<0.01, † p<0.05;

These are negative binomial models for the US as a whole. In the CASE and DEATH model, period effects were entered as a continuous variables (period plus period^2 for cases, just period for deaths), while in the doubling time model period was entered as a categorical variable, similar to a stepped wedge design. The log beta period effect for the CASE models were 0.063 (95% CI 0.05-0.08) and the quadratic is -0.00008. The log beta period for DEATH was -0.092 (-0.11- -0.07). The estimated period effects from the Doubling time model are shown in the graph below (Period effect + Intercept).

1 For the DEATH rate model, excluded variables were not entered into the model due to issues with model convergence. In addition, Census Groups were collapsed into 6 groups: Pacific, Mountain, North Central, South Central (the reference), South Atlantic, and Middle Atlantic/New England.

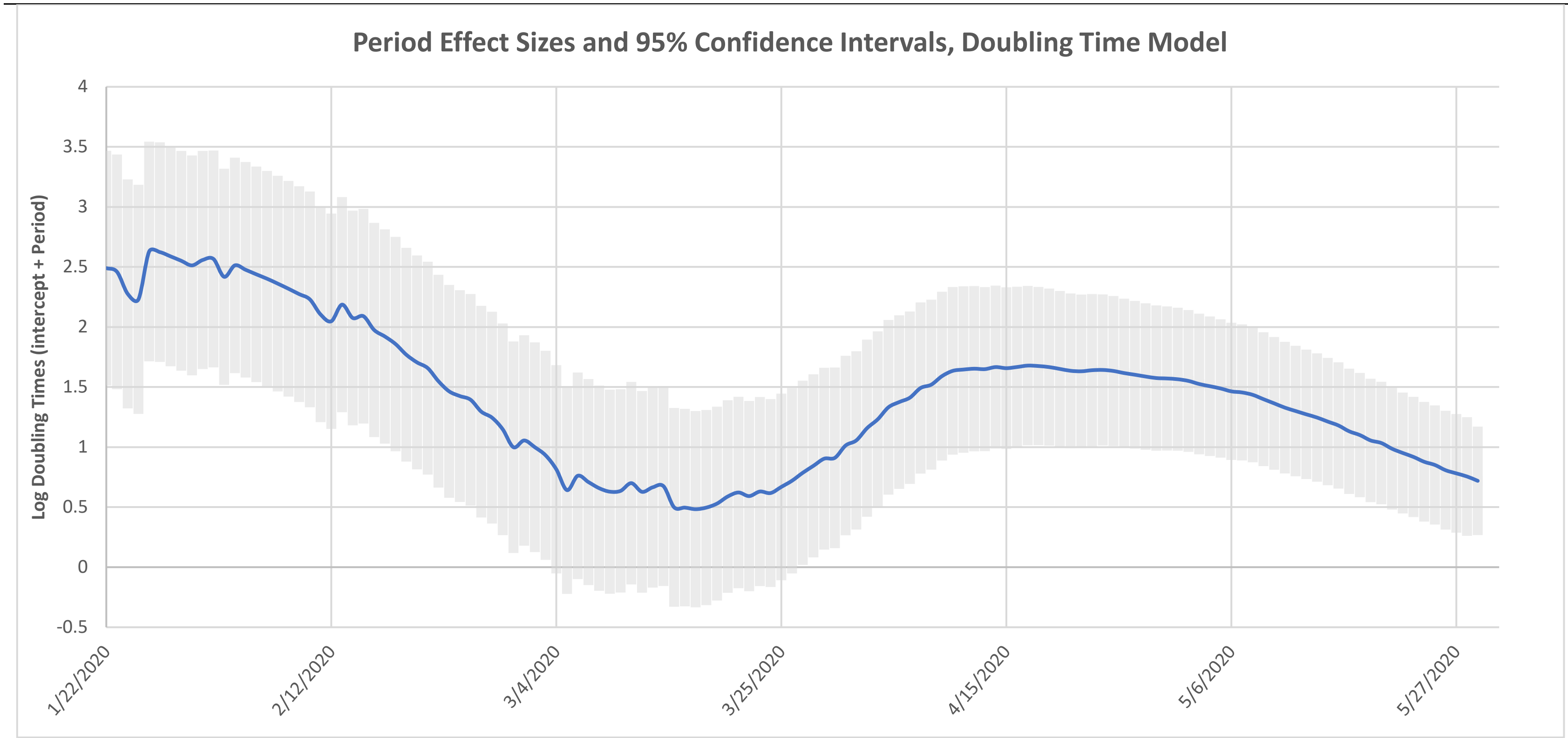

Period Effect Sizes and 95% Confidence Intervals, Doubling Time Model
Log Doubling Times (intercept + Period)
4
3.5
3
2.5
2
1.5
1
0.5
0
-0.5
1/22/2020
2/12/2020
3/4/2020
3/25/2020
4/15/2020
5/6/2020
5/27/2020

Supplemental Table B1. Multivariate models evaluating new case detection by US Census Region

	Pacific States	Mountain States	North Central States	South Central States	South Atlantic States	Mid Atlantic & New Eng.
	Log Beta (95% CI)	Log Beta (95% CI)	Log Beta (95% CI)	Log Beta (95% CI)	Log Beta (95% CI)	Log Beta (95% CI)
**Intercept**	-19.282 (-21.1--17.5) †††	-19.004 (-21.35--16.66) †††	-16.75 (-17.69--15.81) †††	-12.381 (-14.17--10.6) †††	-14.35 (-16.53--12.17) †††	-22.723 (-25.49--19.95) †††
**Period**	0.053 (0.02-0.08) ††	0.054 (0.01-0.1) †	0.059 (0.04-0.08) †††	-0.012 (-0.05-0.02)	0.007 (-0.04-0.05)	0.165 (0.11-0.22) †††
**Period^2**	0.001 (0-0) †††	0.0001 (0-0) †††	-0.0004 (0-0) †††	0.0001 (0-0) †††	0.00004 (0-0) †††	-0.001 (0-0) †††
**Rural-Urban Code (Ref=Code 9)[a]**						
**Code 1**	-0.23 (-1.3-0.84)	0.219 (-0.65-1.09)	0.298 (-0.07-0.67)	0.093 (-0.33-0.52)	-0.143 (-0.67-0.39)	1.557 (0.48-2.63) ††
**Code 2**	-0.529 (-1.54-0.48)	-0.683 (-1.4-0.04)	-0.042 (-0.41-0.33)	-0.176 (-0.56-0.21)	-0.278 (-0.78-0.23)	1.681 (0.63-2.73) ††
**Code 3**	-0.043 (-1.04-0.95)	-0.454 (-1.13-0.23)	0.488 (0.15-0.82) ††	0.036 (-0.36-0.43)	-0.059 (-0.56-0.44)	1.368 (0.31-2.43) †
**Code 4**	-0.383 (-1.43-0.67)	-0.696 (-1.48-0.09)	0.111 (-0.25-0.47)	-0.16 (-0.63-0.31)	-0.323 (-0.88-0.23)	1.088 (0.04-2.14) †
**Code 5**	-0.387 (-1.5-0.72)	-0.327 (-1.1-0.45)	-0.21 (-0.68-0.26)	0.006 (-0.55-0.57)	-0.167 (-1.04-0.7)	1.018 (-0.48-2.52)
**Code 6**	-0.024 (-1.08-1.03)	-0.767 (-1.43--0.11) †	0.212 (-0.08-0.5)	0.017 (-0.32-0.35)	0.156 (-0.33-0.64)	1.139 (0.09-2.19) †
**Code 7**	0.464 (-0.56-1.49)	-0.299 (-0.87-0.27)	-0.103 (-0.41-0.2)	-0.154 (-0.51-0.2)	0.149 (-0.41-0.71)	0.126 (-0.97-1.22)
**Code 8**	0.823 (-0.55-2.2)	-1.239 (-2.3--0.17) †	0.01 (-0.38-0.4)	-0.225 (-0.66-0.21)	0.219 (-0.33-0.77)	0.764 (-0.49-2.02)
**% of 2018 County Population:**						
**… with ≥ Bachelor degree**	0.021 (0-0.04)	0.019 (0.002-0.04) †	-0.007 (-0.02-0.005)	-0.02 (-0.03--0.005) ††	-0.006 (-0.02-0.004)	0.019 (0.004-0.034) †
**… Black (alone or mixed)**	0.016 (-0.05-0.09)	0.042 (-0.05-0.14)	0.025 (0.01-0.04) ††	0.041 (0.03-0.05) †††	0.025 (0.02-0.03) †††	0.041 (0.02-0.07) ††
**… Hispanic (alone or mixed)**	0.022 (0.01-0.04) ††	0.003 (-0.01-0.01)	0.09 (0.08-0.1) †††	0.002 (-0.004-0.01)	0.046 (0.033-0.06) †††	0.055 (0.035-0.07) †††
**… living in Poverty**	-0.033 (-0.08-0.01)	0.068 (0.03-0.1) ††	-0.03 (-0.05--0.01) ††	-0.031 (-0.05--0.01) ††	-0.017 (-0.04-0.01)	-0.069 (-0.12--0.02) ††
**Net county migration rate, 2018**	0.009 (-0.01-0.03)	-0.009 (-0.02-0.01)	0.01 (-0.001-0.02)	-0.004 (-0.01-0.01)	-0.004 (-0.01-0.01)	0.03 (0.003-0.06) †
**Compliance, 10-Day Average**	0.328 (0.24-0.42) †††	0.222 (0.15-0.29) †††	-0.039 (-0.09-0.01)	0.08 (0.02-0.14) †	0.25 (0.19-0.31) †††	0.332 (0.23-0.43) †††
**Policy Intervention (Ref=0, none)[b]**						
**Level 1**	0.959 (0.38-1.53) ††	1.485 (0.71-2.26) ††	1.09 (0.65-1.53) †††	0.424 (-0.1-0.95)	0.214 (-0.23-0.66)	0.574 (0.07-1.07) †
**Level 2**	0.859 (0.13-1.59) †	2.29 (1.45-3.13) †††	1.868 (1.39-2.35) †††	1.42 (0.86-1.98) †††	0.8 (0.29-1.31) ††	1.227 (0.63-1.83) †††
**Level 3**	0.979 (0.22-1.74) †	1.807 (0.98-2.64) †††	2.085 (1.6-2.57) †††	1.294 (0.71-1.88) †††	1.19 (0.65-1.73) †††	1.462 (0.85-2.07) †††
**Level 4**	1.065 (0.32-1.81) ††	2.135 (1.28-2.99) †††	2.138 (1.64-2.64) †††	1.605 (1.01-2.2) †††	1.519 (0.96-2.07) †††	1.359 (0.73-1.99) †††
**Duration of Intervention (days) [b]**						
**Level 1**	0.016 (-0.03-0.06)	0.1 (0.02-0.18) †	0.013 (-0.04-0.07)	0.033 (-0.03-0.1)	0.047 (-0.01-0.1)	0.076 (0.03-0.12) ††
**Level 2**	-0.095 (-0.2-0.01)	-0.2 (-0.27--0.13) †††	-0.021 (-0.07-0.02)	-0.077 (-0.13--0.02) ††	0.061 (0.02-0.1) ††	0.16 (0.09-0.23) †††
**Level 3**	-0.103 (-0.21-0.005)	-0.037 (-0.09-0.0143)	0.049 (0.03-0.07) †††	0.015 (-0.02-0.05)	0.034 (-0.01-0.08)	0.035 (-0.03-0.1)
**Level 4**	-0.147 (-0.18--0.11) †††	-0.047 (-0.1-0.004)	0.028 (0.01-0.05) †	0.01 (-0.02-0.04)	0.001 (-0.04-0.04)	0.037 (-0.02-0.09)
**Compliance (10-day average) * Duration of Intervention (days)**						
**Level 1**	-0.002 (-0.01-0.004)	-0.008 (-0.02-0.002)	0.009 (0.002-0.016) ††	-0.007 (-0.01-0.001)	-0.015 (-0.02--0.008) ††	-0.01 (-0.02--0.001) †
**Level 2**	-0.013 (-0.03-0.003)	0.007 (0-0.014)	0.013 (0.01-0.018) †††	0.012 (0.01-0.019) ††	-0.014 (-0.02--0.008) †††	-0.016 (-0.03--0.007) ††
**Level 3**	-0.013 (-0.02--0.001) †	-0.004 (-0.01--0.002) †††	-0.002 (-0.003--0.001) †††	0.001 (-0.001-0.003)	-0.006 (-0.008--0.004) †††	-0.012 (-0.02--0.005) ††
**Level 4**	-0.002 (-0.003--0.001) †††	-0.003 (-0.004--0.002) †††	-0.001 (-0.001--0.001) †††	-0.002 (-0.002--0.002) †††	-0.001 (-0.001--0.001) ††	-0.004 (-0.004--0.004) †††

†††p<0.0001, †† p<0.01, † p<0.05;

Results are from 6 separate negative binomial models for case rates for each census region. Estimates are log beta incidence rates where negative values indicate reductions in case rates and positive values indicate increases in case rate.

Supplemental Figure B1. Interventions Effects on COVID-19 Cases by US Census Regions

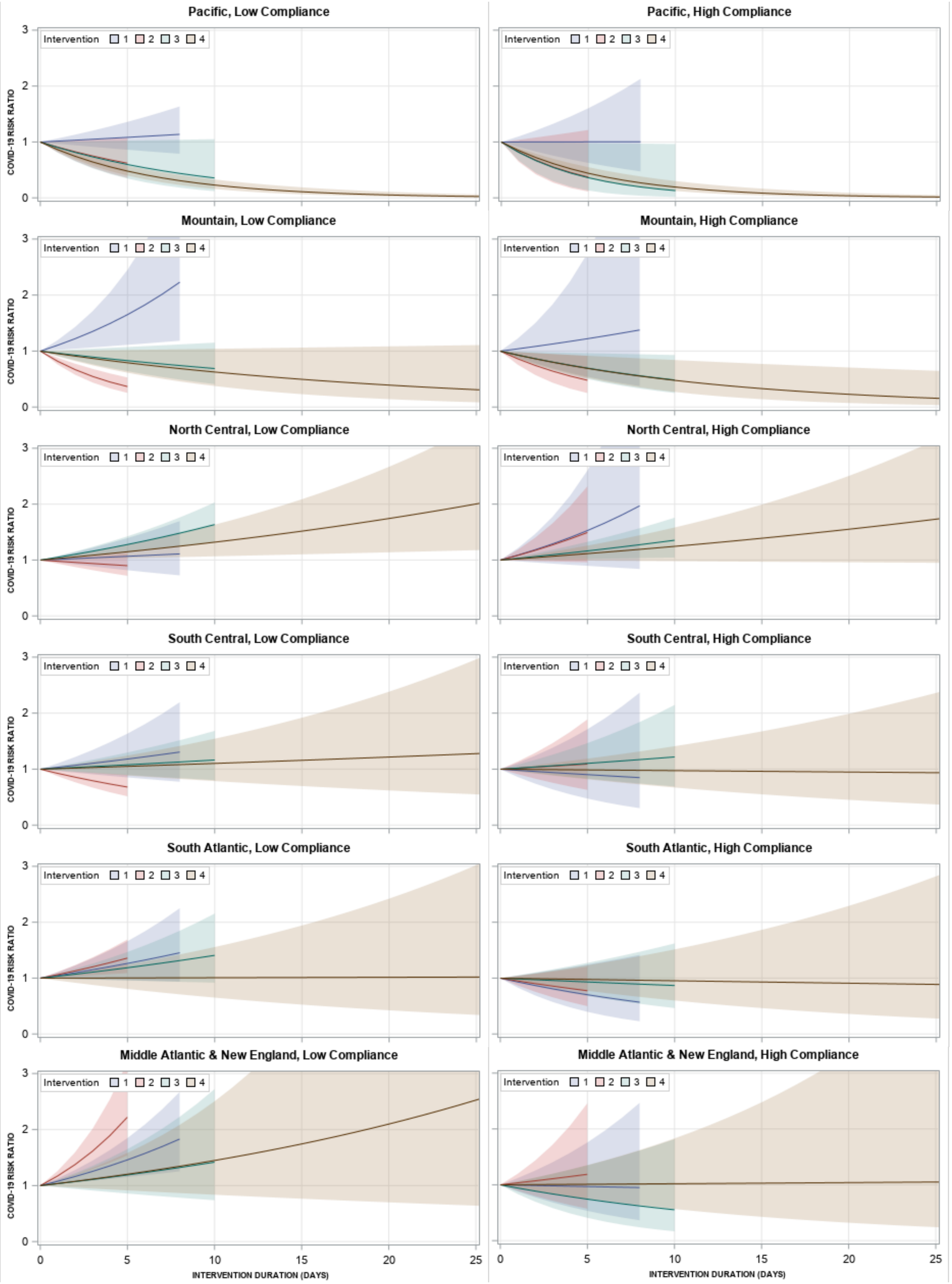

Results are from 6 negative binomial multivariate models, one for each Census Region, shown in Supplemental Table B1. Low compliance sets compliance to 0 over the past 10 days, high compliance sets compliance to 8.

Supplemental Table C1. Doubling Time Models by US Census Region

		Pacific		Mountain		North Central		South Central		South Atlantic		Mid Atlantic/New England	
		**Log Beta**	**(95% CI)**	**Log Beta**	**(95% CI)**	**Log Beta**	**(95% CI)**	**Log Beta**	**(95% CI)**	**Log Beta**	**(95% CI)**	**Log Beta**	**(95% CI)**
**Intercept**		-27.71	(-32.9--22.4)†††	-7.63	(-13.71--1.56)†	1.58	(-0.141-3.29)	4.06	(2.58-5.54)†††	3.63	(1.97-5.29)†††	11.04	(4.35-17.72)††
**Rural-Urban Continuum Code (Ref=9-Rural <2500 people, not adjacent to metro)**	1 - Metro >1 million people	0.095	(-0.546-0.735)	0.641	(0.086-1.196)†	0.455	(0.25-0.66)†††	0.679	(0.46-0.898)†††	0.52	(0.286-0.754)†††	0.128	(-0.356-0.612)
	2 - Metro, 250K - 1 million people	0.249	(-0.367-0.864)	0.794	(0.348-1.24)††	0.45	(0.25-0.65)†††	0.795	(0.602-0.987)†††	0.457	(0.237-0.678)†††	0.076	(-0.395-0.548)
	3 - Metro, <250K people	0.29	(-0.32-0.9)	0.803	(0.384-1.221)††	0.496	(0.314-0.678)†††	0.58	(0.379-0.781)†††	0.484	(0.263-0.704)†††	0.052	(-0.42-0.524)
	4 - Non-metro, >20K people, metro adjacent	0.26	(-0.382-0.903)	0.785	(0.321-1.249)††	0.438	(0.242-0.634)†††	0.725	(0.494-0.957)†††	0.499	(0.255-0.743)†††	0.196	(-0.268-0.661)
	5 -Non-metro, >20K people, metro not adjacent	0.5	(-0.169-1.169)	0.862	(0.388-1.337)††	0.473	(0.22-0.726)††	0.761	(0.484-1.039)†††	0.777	(0.386-1.168)†††	1.002	(0.273-1.731)††
	6 -Non-metro, 2500-19,999, metro adjacent	0.416	(-0.213-1.045)	0.374	(-0.034-0.781)	0.212	(0.057-0.368)††	0.546	(0.376-0.716)†††	0.419	(0.207-0.631)††	0.249	(-0.214-0.712)
	7 -Non-metro, 2500-19,999, metro not adjacent	0.395	(-0.234-1.024)	0.407	(0.047-0.766)†	0.182	(0.02-0.344)†	0.45	(0.269-0.63)†††	0.226	(-0.021-0.473)	0.367	(-0.11-0.844)
	8 - Rural, <2500 people, adjacent to metro	-0.753	(-1.593-0.086)	-0.189	(-0.883-0.506)	0.162	(-0.045-0.368)	0.431	(0.215-0.647)†††	0.085	(-0.156-0.326)	0.679	(0.11-1.249)†
**County Population Density, 2018**		-0.04	(-0.214-0.134)	-0.063	(-0.882-0.756)	0.154	(-0.221-0.529)	0.318	(-0.2-0.836)	-0.148	(-0.268--0.028)†	-0.011	(-0.043-0.022)
**Percent of County Population:**	… with Bachelors degree (or higher)	0.014	(0.001-0.028)†	0.007	(-0.004-0.018)	0.007	(0.001-0.014)†	-0.002	(-0.01-0.006)	0.012	(0.007-0.017)†††	0.022	(0.014-0.03)†††
	… Living in poverty	-0.005	(-0.032-0.023)	-0.01	(-0.031-0.011)	0.007	(-0.004-0.018)	-0.009	(-0.019-0.002)	-0.005	(-0.016-0.005)	0.022	(-0.002-0.047)
	… Hispanic	-0.002	(-0.01-0.006)	-0.002	(-0.008-0.005)	-0.005	(-0.012-0.002)	-0.005	(-0.007--0.002)††	-0.002	(-0.008-0.004)	0.003	(-0.008-0.013)
	… Black	-0.014	(-0.057-0.03)	-0.02	(-0.08-0.041)	-0.014	(-0.025--0.002)†	0.005	(0.001-0.008)††	0.005	(0.002-0.007)††	-0.014	(-0.027--0.001)†
**County Net Migration Rate, 2018**		0.002	(-0.01-0.014)	-0.005	(-0.014-0.003)	0.002	(-0.004-0.007)	0.006	(0.001-0.01)†	0.005	(0.001-0.009)†	0.002	(-0.011-0.016)
**Compliance (reduced travel), average past 10 days**		-0.072	(-0.119--0.024)††	-0.043	(-0.07--0.014)††	0.126	(0.105-0.147)†††	-0.012	(-0.034-0.009)	0.043	(0.025-0.062)†††	-0.101	(-0.142--0.06)†††
**Policy Level (Ref=0 - none)**	Level 1	-0.14	(-0.443-0.162)	0.086	(-0.269-0.44)	-0.606	(-0.866--0.346)†††	-0.119	(-0.372-0.134)	0.138	(-0.074-0.35)	0.527	(0.269-0.785)†††
	Level 2	-0.384	(-0.757--0.012)†	0.329	(-0.124-0.783)	-0.723	(-1.015--0.431)†††	0.288	(0.021-0.555)†	0.15	(-0.1-0.4)	0.483	(0.087-0.88)†
	Level 3	-0.336	(-0.746-0.075)	0.135	(-0.333-0.603)	-0.771	(-1.074--0.468)†††	0.226	(-0.061-0.514)	0.101	(-0.163-0.365)	0.788	(0.374-1.201)††
	Level 4	-0.481	(-0.899--0.064)†	0.211	(-0.261-0.684)	-0.921	(-1.231--0.61)†††	0.25	(-0.042-0.541)	0.155	(-0.113-0.422)	0.488	(0.069-0.906)†
**Duration of Policy, days**	Level 1	0.077	(0.041-0.112)†††	0.001	(-0.045-0.048)	0.144	(0.112-0.177)†††	-0.083	(-0.116--0.049)†††	-0.058	(-0.085--0.032)†††	-0.029	(-0.057-0)†
	Level 2	0.265	(0.197-0.333)†††	-0.029	(-0.11-0.052)	0.022	(-0.007-0.052)	-0.105	(-0.133--0.077)†††	-0.021	(-0.042-0.001)	-0.085	(-0.172-0.003)
	Level 3	0.361	(0.279-0.444)†††	0.116	(0.035-0.197)††	-0.002	(-0.022-0.019)	-0.008	(-0.027-0.011)	0.013	(-0.008-0.034)	-0.208	(-0.297--0.119)†††
	Level 4	0.376	(0.305-0.447)†††	0.129	(0.048-0.21)††	0.014	(-0.006-0.035)	-0.027	(-0.044--0.009)††	-0.024	(-0.045--0.003)†	-0.128	(-0.216--0.04)††
**Compliance x Duration Interaction**	Level 1	0.003	(0.001-0.005)††	-0.004	(-0.008--0.001)†	-0.022	(-0.024--0.02)†††	0.002	(0-0.004)	0.004	(0.002-0.006)†††	-0.001	(-0.003-0.002)
	Level 2	0.008	(0.002-0.014)†	0.011	(0.007-0.02)†††	-0.005	(-0.007--0.003)†††	0.01	(0.008-0.012)†††	-0.004	(-0.005--0.002)†††	0	(-0.003-0.003)
	Level 3	0.011	(0.006-0.016)†††	0	(0-0.001)	0	(0-0)	-0.003	(-0.003--0.002)†††	-0.003	(-0.004--0.003)†††	0.008	(0.006-0.011)†††
	Level 4	0.002	(0.002-0.003)†††	0.001	(0.001-0.001)†††	0	(0-0)	0.001	(0.001-0.001)†††	0	(0-0)†††	0.003	(0.003-0.003)†††

†††p<0.0001, †† p<0.01, † p<0.05

Supplemental Figure C1. Predicted Doubling Time from observed data by duration of intervention for each Census Region with 95% confidence intervals.

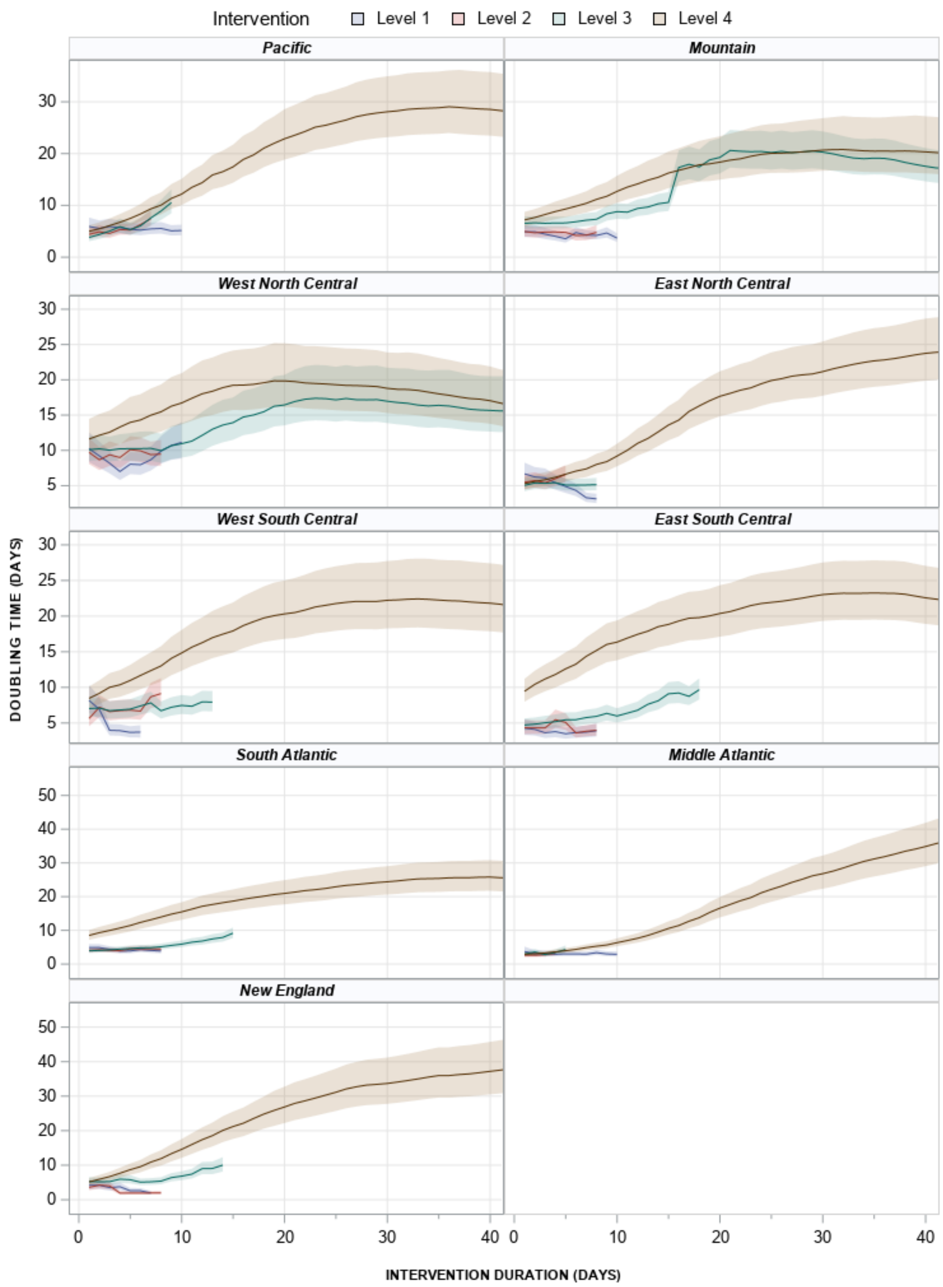

Estimates are mean policy intervention effects for each US region based on model results in Supplemental Table C2 and the observed data from each region. Note that increases in doubling time do not indicate that the policy effect is significant; rather, there are several factors influencing predicted doubling time. The significance of the policy effect is reported in Supplemental Table C1.

Supplemental Table D1. Multivariate Model Results for COVID-19 Death Rates

	PACIFIC		MOUNTAIN		NORTH CENTRAL		SOUTH CENTRAL		SOUTH ATLANTIC		MID ATLANTIC & NEW ENGLAD	
**Variable**	**Log beta**	**SE**	**Log beta**	**SE**	**Log beta**	**SE**	**Log beta**	**SE**	**Log beta**	**SE**	**Log beta**	**SE**
Intercept	-7.637	2.264††	-30.996	32.244	-26.706	13.28†	-10.767	9.197	0.82	5.558	-19.673	12.446
Period	0.038	0.043	0.475	0.634	0.339	0.256	0.066	0.164	-0.155	0.109	0.257	0.238
County Poverty %	-0.022	0.104	0.021	0.178	0.168	0.085†	0.032	0.075	0.091	0.046	-0.014	0.093
Compliance	-0.485	0.37	-1.827	1.564	-0.253	0.494	0.359	0.548	-0.803	0.76	0.241	0.996
Policy Level 2	1.074	0.711	0.493	1.624	0.276	1.205	-1.122	1.263	1.635	0.824†	-1.52	1.125
Policy 2 Duration	-0.546	0.283	-0.367	0.691	-0.471	0.448	0.295	0.315	-0.154	0.165	-0.054	0.324
Compliance*Duration	0.208	0.108	0.082	0.167	-0.003	0.087	-0.07	0.082	0.142	0.104	0.013	0.123
Intercept	-2.747	0.251†††	-2.419	0.582†††	-3.075	0.115†††	-2.465	0.725††	0.25	0.564	-1.96	0.586††
Period	-0.053	0.005†††	-0.007	0.011	0.005	0.002††	-0.005	0.013	-0.065	0.01†††	-0.031	0.011††
County Poverty %	0.184	0.013†††	0.04	0.006†††	0.038	0.002†††	0.017	0.004†††	0.062	0.004†††	0.057	0.009†††
Compliance	0.142	0.045††	-0.051	0.017††	-0.019	0.007†	-0.05	0.025†	0.016	0.021	-0.228	0.069††
Policy Level 3	-0.703	0.247††	-0.731	0.117†††	0.164	0.063††	-0.115	0.096	-0.312	0.087††	-0.483	0.13††
Policy 3 Duration	0.185	0.149	-0.015	0.012	-0.031	0.002†††	0.07	0.022††	0.067	0.015†††	0.117	0.036††
Compliance*Duration	-0.02	0.023	0.002	0.001††	-0.002	0.0002†††	-0.011	0.003†††	-0.012	0.002†††	0.005	0.006
Intercept	-6.257	0.977†††	-4.38	0.689†††	-4.677	0.295†††	-4.217	0.519†††	-4.378	0.613†††	-2.816	0.878††
Period	0.004	0.018	-0.041	0.01†††	-0.029	0.002†††	-0.032	0.008†††	-0.02	0.01†	-0.057	0.014†††
County Poverty %	0.021	0.022	0.034	0.012††	0.016	0.009	0.024	0.006††	0.004	0.006	-0.019	0.017
Compliance	-0.023	0.036	-0.038	0.041	-0.001	0.016	0.015	0.019	-0.045	0.016††	0.023	0.02
Policy Level 4	0.272	0.309	0.849	0.229††	0.784	0.132†††	0.337	0.122††	0.255	0.108†	0.442	0.226†
Policy 4 Duration	-0.057	0.021††	-0.002	0.014	-0.008	0.003†	-0.004	0.008	-0.022	0.01†	0.03	0.015†
Compliance*Duration	0.002	0.001	0.001	0.001	0.001	0.0003†	-0.001	0.0004	0.002	0.0004†††	0.001	0.0004††

†††p<0.0001, †† p<0.01, † p<0.05

Results from this table are from three county-level models for each US Census Region (one for each intervention level). Period refers to the study day (day 1=January 22, 2020); Data for each model are constrained such that the policy effect is only comparing the specific level to all lower level policies. For example, the model for Policy Level 3 includes an indicator for policy level 3 and this is compared policies 0-2.

Supplemental Figure D1. Climate Parameter Effects on COVID-19 Doubling Time by US Census Region

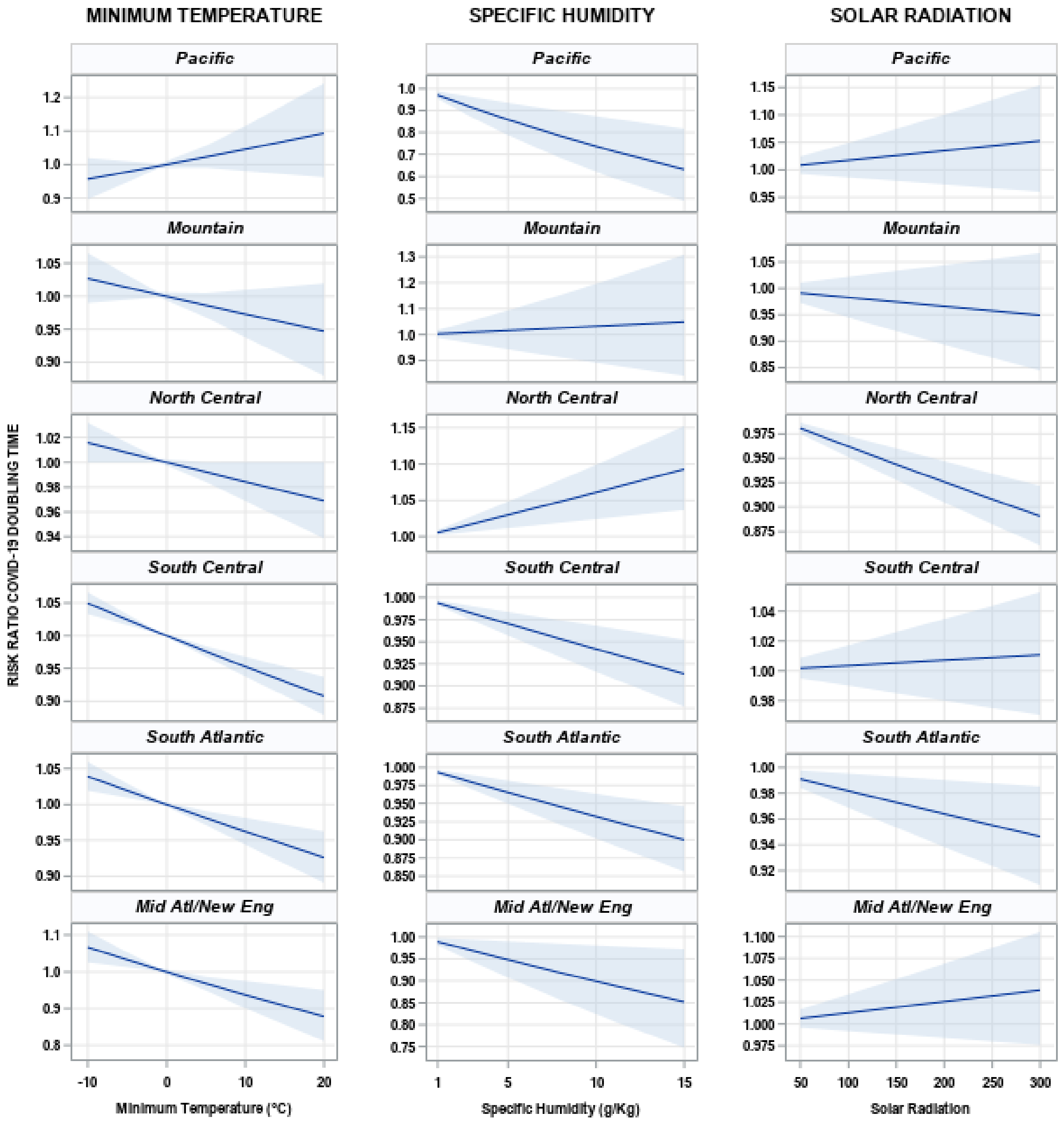

Results are from 3 separate models, one for each lagged climate variable, estimating the doubling time of cases, adjusting for period, rural-urban classification, percent of the county population with a bachelor degree or higher, compliance with policy, US Census Region classification, Intervention and duration of intervention. The climate effect and 95% CI for each region are estimated from predicted log-beta values from the overall effect plus Census Region interaction.

Supplemental Figure D2. Climate Parameter Effects on COVID-19 Deaths by US Census Region

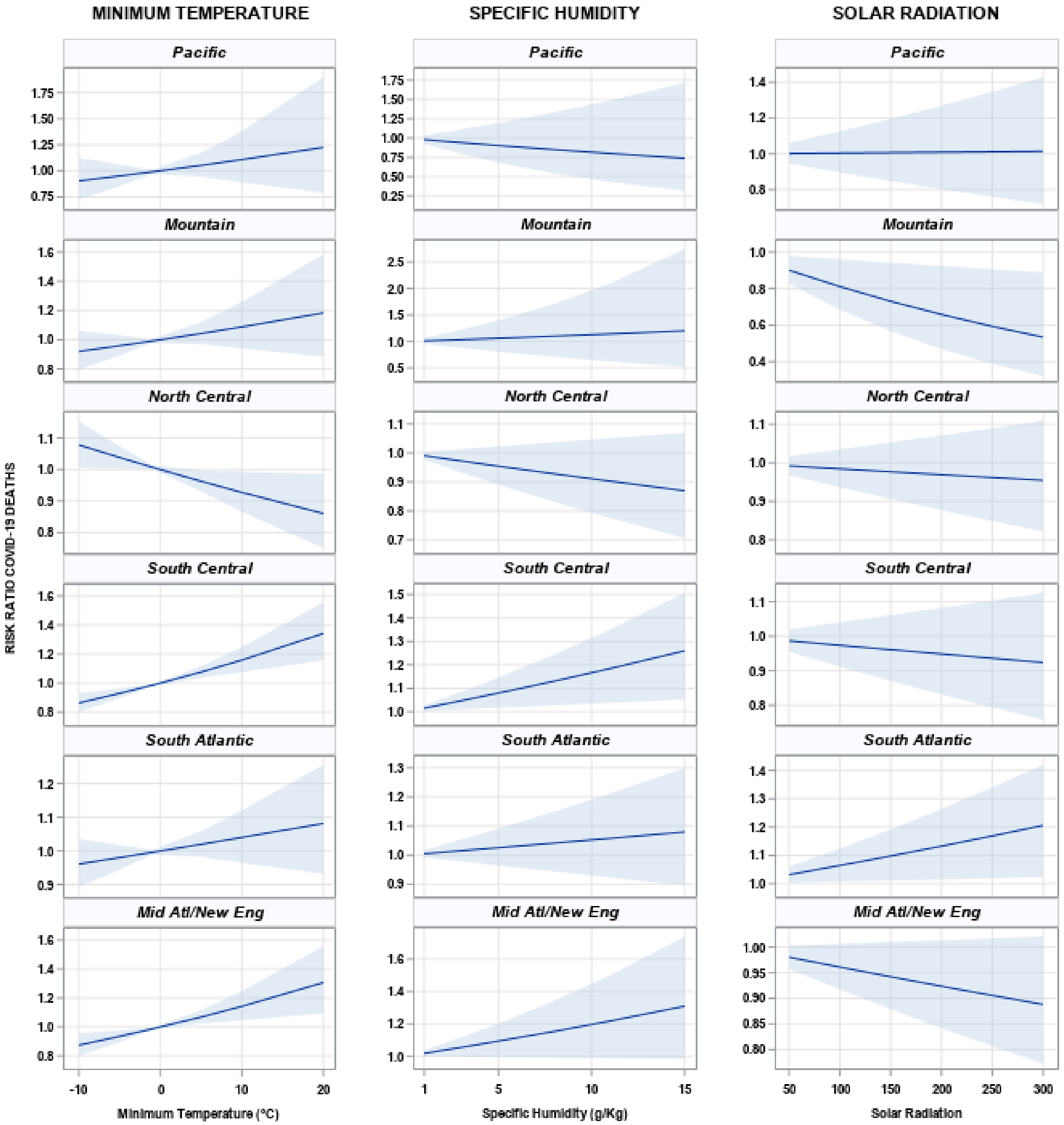

Results are from three separate models, one for each lagged climate variable, estimating the number of deaths, adjusting for period, poverty, compliance, US Census Region, Intervention level and duration of intervention by census group. The overall Type 3 test of fixed effects F-value (and p-value) for minimum temperature is 5.25 (p<0.0001), specific humidity 2.14 (p=0.0454), and solar radiation 2.43 (p=0.0236). The climate effects and 95% CI for each region are estimated from the predicted log-beta values from the overall effect plus the Census Region interaction.
